# Genome-wide association study of signature genetic alterations among *pseudomonas aeruginosa* cystic fibrosis isolates

**DOI:** 10.1371/journal.ppat.1009681

**Published:** 2021-06-23

**Authors:** Wontae Hwang, Ji Hyun Yong, Kyung Bae Min, Kang-Mu Lee, Ben Pascoe, Samuel K Sheppard, Sang Sun Yoon

**Affiliations:** 1 Department of Microbiology and Immunology, Seoul, Republic of Korea; 2 Brain Korea 21 PLUS Project for Medical Sciences, Seoul, Republic of Korea; 3 The Milner Centre for Evolution, Department of Biology and Biochemistry, University of Bath, Claverton Down, Bath, United Kingdom; 4 Institute for Immunology and Immunological Diseases, Seoul, Republic of Korea; 5 Severance Biomedical Science Institute, Yonsei University College of Medicine, Seoul, Republic of Korea; Columbia University, UNITED STATES

## Abstract

*Pseudomonas aeruginosa* (PA) is an opportunistic pathogen that causes diverse human infections including chronic airway infection in patients with cystic fibrosis (CF). Comparing the genomes of CF and non-CF PA isolates has great potential to identify the genetic basis of pathogenicity. To gain a deeper understanding of PA adaptation in CF airways, we performed a genome-wide association study (GWAS) on 1,001 PA genomes. Genetic variations identified among CF isolates were categorized into (i) alterations in protein-coding regions, either large- or small-scale, and (ii) polymorphic variation in intergenic regions. We introduced each CF-associated genetic alteration into the genome of PAO1, a prototype PA strain, and validated the outcomes experimentally. Loci readily mutated among CF isolates included genes encoding a probable sulfatase, a probable TonB-dependent receptor (PA2332~PA2336), L-cystine transporter (YecS, PA0313), and a probable transcriptional regulator (PA5438). A promoter region of a heme/hemoglobin uptake outer membrane receptor (PhuR, PA4710) was also different between the CF and non-CF isolate groups. Our analysis highlights ways in which the PA genome evolves to survive and persist within the context of chronic CF infection.

## Author summary

Understanding how human pathogens, such as *Pseudomonas aeruginosa* (PA), adapt to the host environment during prolonged infections can provide important information for the treatment of disease. In this study, we compared the genome sequences of PA isolates from cystic fibrosis (CF) and non-CF patients and identified genetic variation that was significantly associated with CF disease. CF-associated genetic variations included: (i) the deletion of three amino acids in a transcriptional regulator (PA5438), which leads to decreased antibiotic susceptibility, enhanced virulence expression and sustained biofilm production in the presence of transferrin, and (ii) the insertion of three amino acids in a cystine transporter (PA0313), which results in decreased pyochelin production. Furthermore, single nucleotide replacement in a promoter was identified and shown to upregulate the cellular process for iron acquisition. Our bioinformatics analyses, linked with experimental validation via genetic manipulation and phenotypic investigation of mutants, provides novel insights into the genomic plasticity of PA during chronic CF infection.

## Introduction

*Pseudomonas aeruginosa* (PA) is an ubiquitous gram-negative bacterium that can cause disease in plants and animals [1]. PA infection can cause acute syndromes such as pneumonia and bloodstream infections, as well as chronic airway infections in patients with cystic fibrosis (CF). CF is a well-known genetic disorder caused by a mutated cystic fibrosis transmembrane conductance regulator (CFTR) protein. CFTR disruption alters the condition of the lung such that the increasingly dehydrated viscous mucus layer provides a favorable habitat for several pathogens, such as PA, which can infect opportunistically and persist over an extended period of time [2].

The ecological versatility of PA is thought to be associated with its relatively large genome containing numerous regulatory genes that confer an advantage in adapting during prolonged infections [3]. Within-host mutation of PA isolates from a single CF patient have been analyzed to better understand the underlying genetics of adaptation [4]. Expression studies have also been performed on PA grown in CF sputum and have identified altered expression of genes encoding amino acid biosynthesis and degradation, and quinolone signaling [5,6]. The *lasR* gene, which encodes an important quorum-sensing regulator, has frequently been detected among isolates from CF patients [7,8], however, the significance of this mutation is unclear. Mutations leading to increased antibiotic tolerance have also been identified in PA inhabiting the CF airway [9–11]. Furthermore, PA clonal lineages vary across different CF patients, depending on the sputum composition [12–15], and even within an individual patient over time [16,17]. Together, these findings suggest that PA is an adaptable organism that responds flexibly to changing environments.

Most large-scale genetic studies have focused principally on CF isolates. Therefore, in the absence of a comparator non-CF PA population, it is difficult to identify specific genetic variations that are associated with the CF environment. In this study, we compared 1,001 PA genomes sampled from both CF and non-CF clinical isolates. Genome-wide association studies (GWASs) have been useful in uncovering causal relationships between genetic variations and disease phenotypes in human populations [18], with over 4,000 human GWAS conducted to date [19]. In contrast to human genomes, bacterial genomes of the same species can vary in size, gene repertoire, and gene arrangement [20]. Because of this genomic variability, additional considerations must be made when performing bacterial GWAS [21–25]. In this study, we selected the genomes of PA isolates of known origin and performed a GWAS based on k-mer counting [22,23,26], a modified method that permits association mapping of genes and intergenic regions of the PAO1 reference genome. Results provided here expand our current understanding of how gene-level changes correlate with mechanisms of PA adaptation to the CF lung environment.

## Results and discussion

### P. aeruginosa population structure

**Fig 1:**
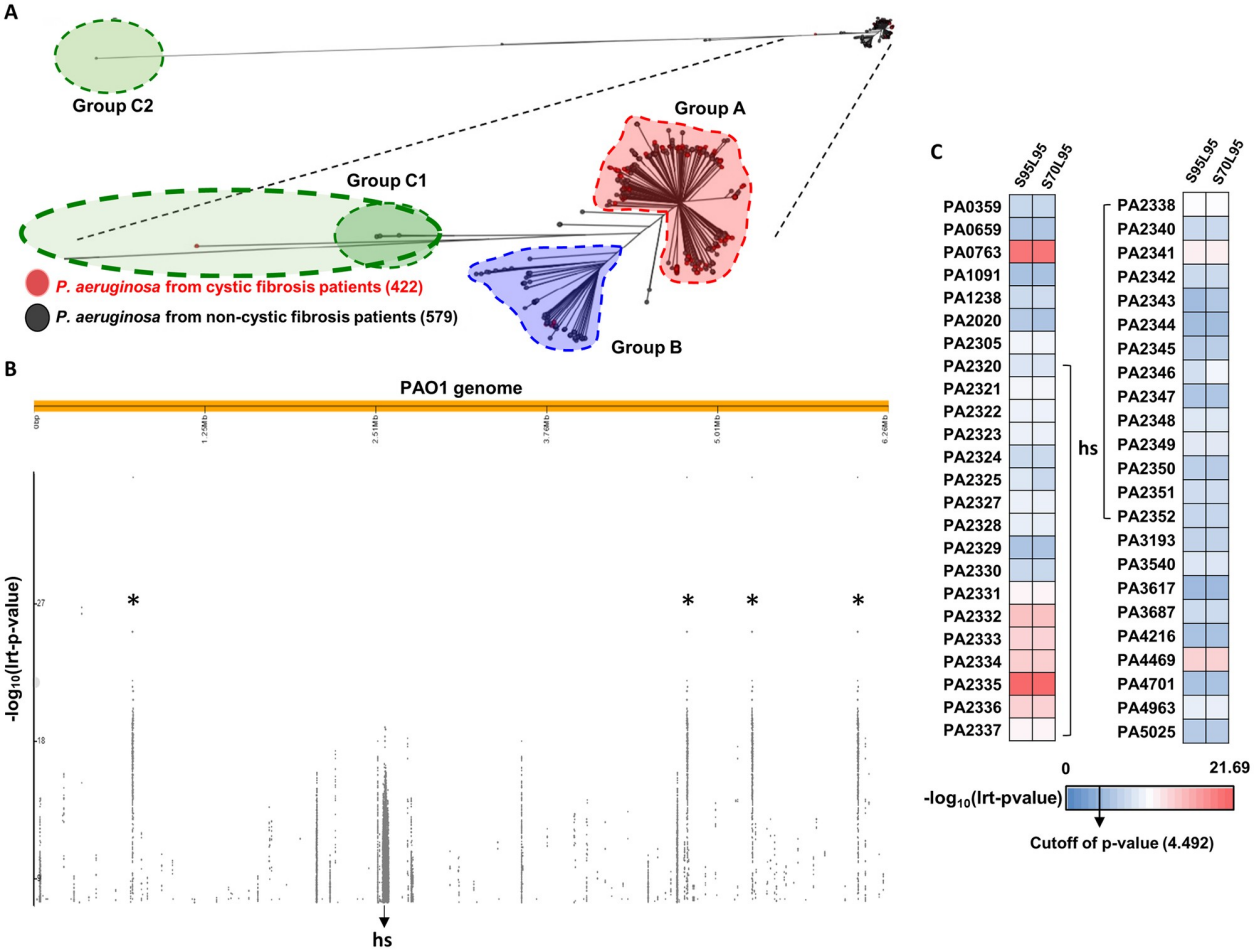
Visualization of significantly different regions and proteins with large-scale mutation. **(A)** Phylogenetic tree was constructed with 1,001 genomes with known host disease information. Black and red leaves each indicate non-CF and CF isolates, and the numbers of CF and non-CF genomes for constructing the phylogenetic tree are placed inside brackets. Most of the CF isolates are located in group A, which includes the majority of genomes, and the smaller groups are designated group B, C1 and C2. **(B)** 31-mers that were significantly associated with CF and non-CF groups were aligned to the whole PAO1 genome and visualized by Phandango. Regions marked by asterisks are ribosomal DNA sequences and ‘hs’ marks the hotspot region where a large number of candidate 31-mers align. The higher the point representing a 31-mer, the more significant the lrt p-value. **(C)** Proteins with large-scale mutation from the PAO1 genome and Lrt p-values calculated for clustering performed under two conditions (S95L95: 95% similarity and 95% length coverage; S70L95: 70% similarity and 95% length coverage) are described in the first and second columns as per the color index beneath the heatmap. ‘hs’ indicates candidate proteins located within the hotspot region in **(B)**.

Most studies aimed at better understanding chronic PA infection in CF patients have focused only on CF isolates [14,16]. It is therefore unclear whether genetic variations highlighted in these studies are important in the context of adaptation to the CF lung. Here, we assembled a collection of 2,167 PA genomes from the Pseudomonas Genome Database [27], including genomes from both CF and non-CF individuals. Genomes of unknown origin were removed and a phylogeny was constructed using 1,001 genomes of known origin (Fig 1A). In order to remove clonal isolates that may increase any potential confounding lineage effects in the subsequent GWAS, the dataset was trimmed to 636 genomes (S1 Fig, S1 Data), maintaining 99.8% diversity of original selection and the overall structure of the phylogenetic tree. Isolates from CF patients could be identified from all parts of the phylogeny, including the previously described groups A and B; and the long branching groups C1 and C2 [28]. Most CF isolates (409 among 422) belonged to group A.

### Identification of CF-associated genetic elements

In total, 636 genomes were used to perform a GWAS using Pyseer [29]. These comprised 206 genomes from CF and 430 from non-CF. A k-mer based approach was implemented to include variability within intergenic regions. We selected k-mers with a length of 31 base pairs (31-mer) consistent with other studies that have adopted similar approaches and tested the effect of changes in k-mer length on GWAS sensitivity and false positive associations [22,23]. Significantly associated k-mers (p-value cutoff = 2.8e-08) were aligned to the PAO1 reference genome and visualized using Phandango [30] (Fig 1B).

GWAS analysis identified 41,685 k-mers associated with CF with a p-value < 2.8e-08. Of these, 489 *de-novo* assembled contigs mapped to 109 protein-coding genes, six untranslated regions and 29 intergenic regions in the PAO1 reference genome. Four most meaningful peaks marked with asterisks in Fig 1B are rDNA sequences. A greater number of CF isolates did not seem to have 16S and 23S rDNA sequences compared with the non-CF group. However, we verified that several of these CF isolates (AU2342, AU6462, AU9739, AU10409, AU16821 and AU25116) do have 16S rDNA by PCR-amplifying and sequencing the 16S rDNA regions. By extension, we believe that the lack of detection of 16S and 23S rDNA sequences from several CF isolates have arisen from an *in silico* error. Despite this, the number of genes annotated using Prokka [31] were not significantly different between genomes and they were therefore included in the input database.

### GWAS elements associated with allelic variation in P. aeruginosa from CF patients

Focusing on the k-mers that were mapped to the PAO1 reference genome, we obtained amino acid sequences for each gene from all isolate genomes. Two different homology clustering thresholds were used: 95% similarity and 95% gene length coverage (S95L95) and 70% similarity and 95% gene length coverage (S70L95) (S2 and S3 Data). GWAS results were then filtered by allele frequency using the likelihood-ratio test (lrt) p-values in Pyseer for both conditions (Fig 1C). This provided a list of candidate genes with multiple associated alleles. We hereinafter refer to these genes with multiple co-localized mutations to contain large-scale mutations. Variation in MucA (PA0763) served as a positive control, as MucA mutation is frequently observed in CF isolates [32,33].

### Large-scale alterations in protein-coding regions

**Fig 2:**
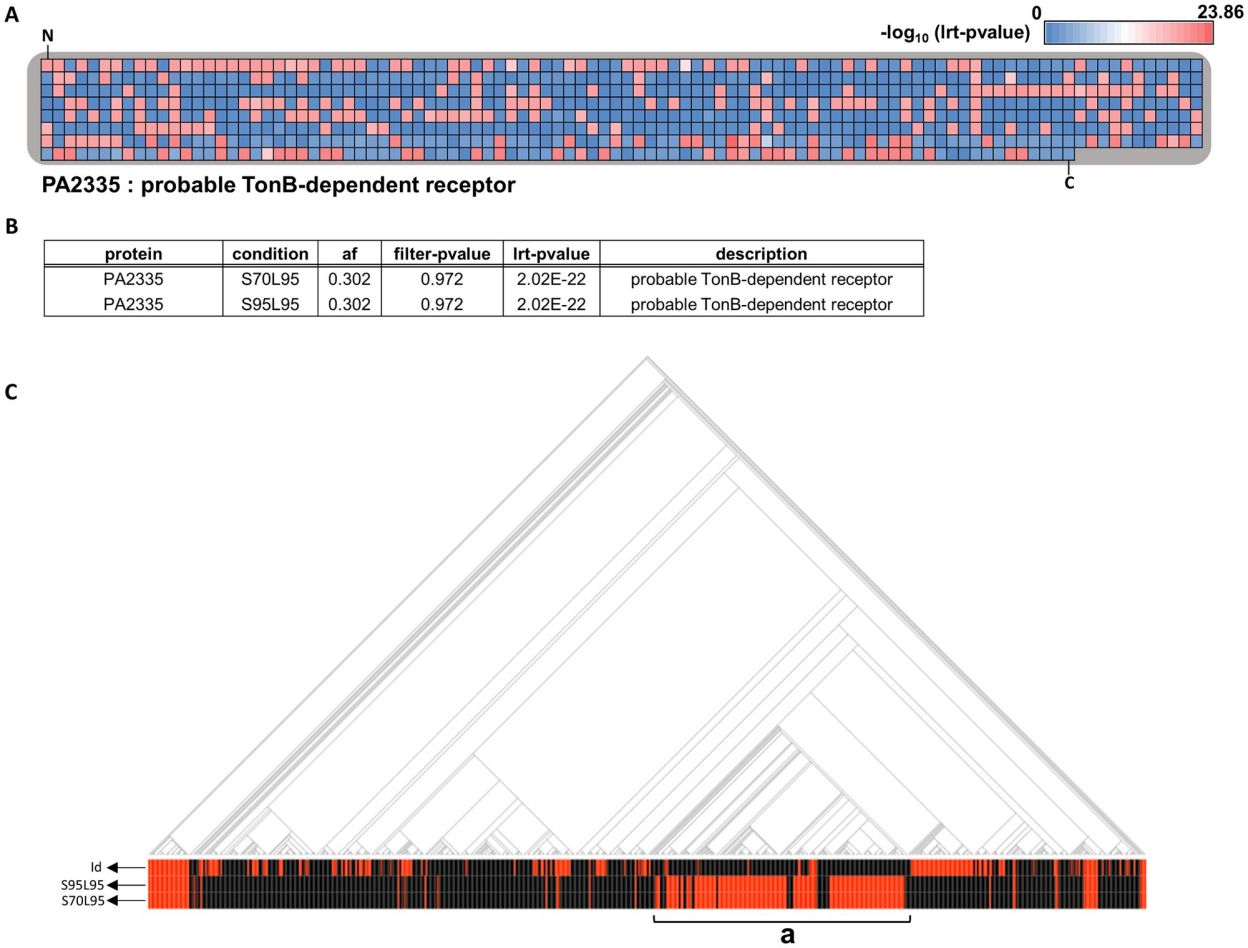
PA2335 candidate with the lowest lrt p-value in the large-scale mutation group. **(A)** Total amino acid residues of PA2335 are presented and each amino acid locus is represented by individual squares. The color of each square indicates the lrt p-value assigned to that amino acid residue based on Pyseer results and colors are assigned according to the index above the heatmap, which was generated based on the negative logs of the maximum and minimum lrt p-values from the Pyseer result of amino acid locus. ‘N’ and ‘C’ mark the N- and C-terminus of PA2335, respectively. **(B)** Pyseer result of the PA2335 cluster is shown. Different conditions used in clustering and the frequency of the PA2335 large alteration are described in ‘condition’ and ‘af’ columns. Population structure is not considered in the calculation of filter p-values, but it is for the calculation of lrt p-values. **(C)** The phylogenetic tree of 636 genomes and their associated disease status are shown. Red and black lines in the ‘id’ row indicate genomes isolated from CF and non-CF patients, respectively. Black lines in the second and third rows indicate that the homolog of PA2335 clustered with the PAO1 protein at given clustering thresholds (S95L95:S70L95). Red lines indicate that the homolog did not cluster with the PAO1 protein. Genomes in region **a** are included in group B indicated in S1 Fig.

Most candidate associated genes were identified within the prominent hs region (Fig 1C), with the most significant lrt p-value (p-value = 2.02e-22) associated with a probable TonB-dependent receptor (S2 Data). Differences in the strength of association were noted across amino acid residues for PA2335 (Fig 2A). Analysis of raw filter p-values seemed to suggest an opposing conclusion in relation to the importance of PA2335 alteration in PA adaptation to the CF airway. However, these raw p-values are not weighted to account for the population structure (Fig 2B and 2C). Mapping variations in PA2335 against the phylogeny demonstrated alignment of the CF phenotype with the presence of its homolog, except for region **a** (Fig 2C). This region corresponds to the group B isolates (S1 Fig) and suggests that these isolates possess alternative CF associations. This pattern was also seen for variants in the PA2333, PA2334, and PA2336 genes. Variation in PA2332 was associated with the CF phenotype across the entire population suggesting it may have a broad influence in CF adaptation (S2 Data). This is consistent with a previous study that identified lineage-specific markers for groups A and B, including genes PA2333~PA2336 which were over-represented in group A [28].

Functional annotation of proteins with large-scale mutations was performed using BlastKOALA [34]. KEGG ontology (KO) [35], definition, and pathway information of the candidate associated genes were identified in the CF or non-CF isolates (Table 1, S3 Data). Almost all of the large-scale mutations were more significantly associated with PA from CF patients. Large scale mutations of MucA (PA0763) are well known in CF isolates to result in increased alginate production and thus contribute to the mucoid phenotype, promoting infection [32,33,36,37]. PA2020 is a negative regulator of the MexXY efflux pump, which expels antibiotics from the cell [38]. Increased MexXY expression level and antibiotic resistance are induced by disruption to the PA2020 regulator. Moreover, CF sputum contains mucins, free amino acids, lactate, and glucose as a potential energy source and PA preferentially uses organic and amino acids over glucose as an energy source [39]. For this reason, we speculate that some CF isolates have evolved with mutations in glucose catabolism genes (PA2321 and PA3193) to avoid spending unnecessary energy in catabolizing a less optimal carbon source (glucose). Additionally, mannitol has been used as a therapeutic treatment to increase mucociliary clearance in the airway and simultaneously induce tobramycin sensitivity in PA by generating a proton motive force [40]. Therefore, we postulate that exposure through the concurrent therapeutic use of mannitol and antibiotics in CF patients may be the background from which the mutations arose in the gene cassette (PA2338, PA2340, PA2341, and PA2342). These large-scale mutations are highlighted in bold in Table 1.

**Table 1:** KEGG annotation of candidate proteins with large-scale mutation. All large-scale mutations were annotated by Blastkoala. Proteins with KEGG information are listed in the ‘Query’ column, and KEGG ontology (KO), definition, and related pathway information are shown. Proteins discussed in the results are highlighted in bold.

Query	KO	Definition	Pathway
**PA0763**	**K03597**	**sigma-E factor negative regulatory protein RseA**	
PA1091	K20444	O-antigen biosynthesis protein	
**PA2020**	**K18129**	**TetR/AcrR family transcriptional regulator, mexXY operon repressor**	**Beta-Lactam resistance**
PA2320	K06145	LacI family transcriptional regulator, gluconate utilization system Gnt-I transcriptional repressor	
**PA2321**	**K00851**	**gluconokinase**	**Pentose phosphate pathway**
PA2322	K03299	gluconate:H+ symporter, GntP family	
PA2323	K00131	glyceraldehyde-3-phosphate dehydrogenase (NADP+)	Glycolysis / Gluconeogenesis, Pentose phosphate pathway
PA2327	K02050	NitT/TauT family transport system permease protein	
PA2328	K02051	NitT/TauT family transport system substrate-binding protein	
PA2329	K02049	NitT/TauT family transport system ATP-binding protein	
PA2335	K02014	iron complex outer membrane recepter protein	
**PA2338**	**K10227**	**sorbitol/mannitol transport system substrate-binding protein**	**ABC transporters**
**PA2340**	**K10229**	**sorbitol/mannitol transport system permease protein**	**ABC transporters**
**PA2341**	**K10111**	**multiple sugar transport system ATP-binding protein**	**ABC transporters**
**PA2342**	**K00045**	**mannitol 2-dehydrogenase**	**Fructose and mannose metabolism**
PA2343	K00854	xylulokinase	
PA2344	K00847	fructokinase	Fructose and mannose metabolism
PA2345	K17218	sulfide:quinone oxidoreductase	Sulfur metabolism
PA2348	K20938	long-chain alkane monooxygenase	
PA2349	K02073	D-methionine transport system substrate-binding protein	ABC transporters
PA2350	K02071	D-methionine transport system ATP-binding protein	ABC transporters
PA2351	K02072	D-methionine transport system permease protein	ABC transporters
PA2352	K01126	glycerophosphoryl diester phosphodiesterase	Glycerophospholipid metabolism
**PA3193**	**K00845**	**glucokinase**	**Glycolysis / Gluconeogenesis**
PA3540	K00066	GDP-mannose 6-dehydrogenase	Fructose and mannose metabolism
PA3617	K03553	recombination protein RecA	Homologous recombination
PA3687	K01595	phosphoenolpyruvate carboxylase	Pyruvate metabolism
PA4216	K20262	dihydrophenazinedicarboxylate synthase	Phenazine biosynthesis, Quorum sensing
PA4701	K07028	uncharacterized protein	
PA4963	K09857	uncharacterized protein	
PA5025	K01740	O-acetylhomoserine (thiol)-lyase	Cysteine and methionine metabolism

### Small-scale alterations in protein-coding regions

Large-scale mutations are more likely to cause loss-of-function than scattered substitutions in homologous sequence (small-scale mutation). However, adaptation to the CF lung may also involve substitutions that alter protein-coding regions leading to additive effects. To investigate this, we analyzed the top 10 amino acid residues of the small-scale mutations (Tables 2 and 3). For the genes containing the most significant CF-associated variation, the functional genomics was further analyzed with laboratory validation experiments.

**Table 2:** Top 10 amino acid residues of the small-scale mutation group with 31-mers aligned to the PAO1 genome. Top 10 amino acid residues according to Pyseer results with 31-mers aligned to the PAO1 genome are presented. The reference protein corresponding to each residue is presented in the ‘protein’ column, and the ‘locus’ column contains the locus of the mutation and amino acid residue of the reference protein where the mutation is detected. SLR sequences of PA5438 are highlighted in bold. Multiple types of mutation at each locus of the reference protein may be present and detailed information is provided in S3 Data.

protein	locus	af	filter-pvalue	lrt-pvalue	description
**PA5438**	**272S**	**0.075**	**7.03E-20**	**9.92E-17**	**Probable transcriptional regulator**
**PA5438**	**273L**	**0.079**	**1.20E-19**	**4.41E-15**	**Probable transcriptional regulator**
**PA5438**	**274R**	**0.08**	**6.33E-19**	**3.36E-14**	**Probable transcriptional regulator**
PA4914	73A	0.05	2.71E-14	7.30E-13	Transcriptional regulator, AmaR
PA3355	384D	0.05	2.71E-14	3.48E-12	Hypothetical protein
PA2354	362G	0.09	3.36E-13	2.27E-11	SfnR1
PA2354	369E	0.08	2.20E-14	3.33E-11	SfnR1
PA2354	370R	0.08	2.20E-14	3.33E-11	SfnR1
PA2354	371A	0.08	2.20E-14	3.33E-11	SfnR1
PA2354	373R	0.08	2.20E-14	3.33E-11	SfnR1

**Table 3:** Top 10 amino acid residues of the small-scale mutation group whose 31-mers did not align to the PAO1 genome. Top 10 amino acid residues according to Pyseer results whose 31-mers did not align to the PAO1 genome are shown. Reference proteins from genomes other than PAO1 and the PAO1 homolog of this reference protein are presented in columns ‘protein’ and ‘homolog’. The ‘locus’ indicates the mutation and amino acid of the reference protein where the mutation is detected. Multiple types of mutation at each locus of the reference protein may be present and detailed information is provided in S3 Data. The reference protein name in the “protein” column is composed of the genome id, connected by the latter underscore sign, to gene number within that genome. All sequences of the reference proteins in Table 3 are provided in S4 Data.

Protein	locus	af	filter-pvalue	lrt-pvalue	homolog (PAO1)	description
**AU17965_3981_04951**	**162S**	**0.0708**	**1.27E-19**	**3.80E-16**	**PA0313**	**L-cystine transporter of ABC system YecS**
**AU17965_3981_04951**	**163L**	**0.0708**	**1.27E-19**	**3.80E-16**	**PA0313**	**L-cystine transporter of ABC system YecS**
**AU17965_3981_04951**	**164I**	**0.0708**	**1.27E-19**	**3.80E-16**	**PA0313**	**L-cystine transporter of ABC system YecS**
105738_3985_01941	57G	0.95	2.71E-14	1.12E-12	PA1384	UDP-glucose 4-epimerase
AZPAE14712_2411_01394	206G	0.928	2.19E-16	1.38E-09	PA2353	Conserved hypothetical protein
AZPAE14712_2411_01394	198P	0.0739	1.01E-15	4.72E-09	PA2353	Conserved hypothetical protein
AZPAE14712_2411_01394	200R	0.0739	1.01E-15	4.72E-09	PA2353	Conserved hypothetical protein
AZPAE15072_2259_00589	283T	0.0503	2.71E-14	1.70E-08	PA3848	Hypothetical protein
AZPAE15072_2259_00888	176D	0.0503	2.71E-14	1.70E-08	PA3594	Probable transcriptional regulator
AZPAE15072_2259_02084	305K	0.0503	2.71E-14	1.70E-08	PA0242	Hypothetical protein

### Mutations in a putative transcriptional regulator (PA5438)

#### Identification and experimental validation

**Fig 3:**
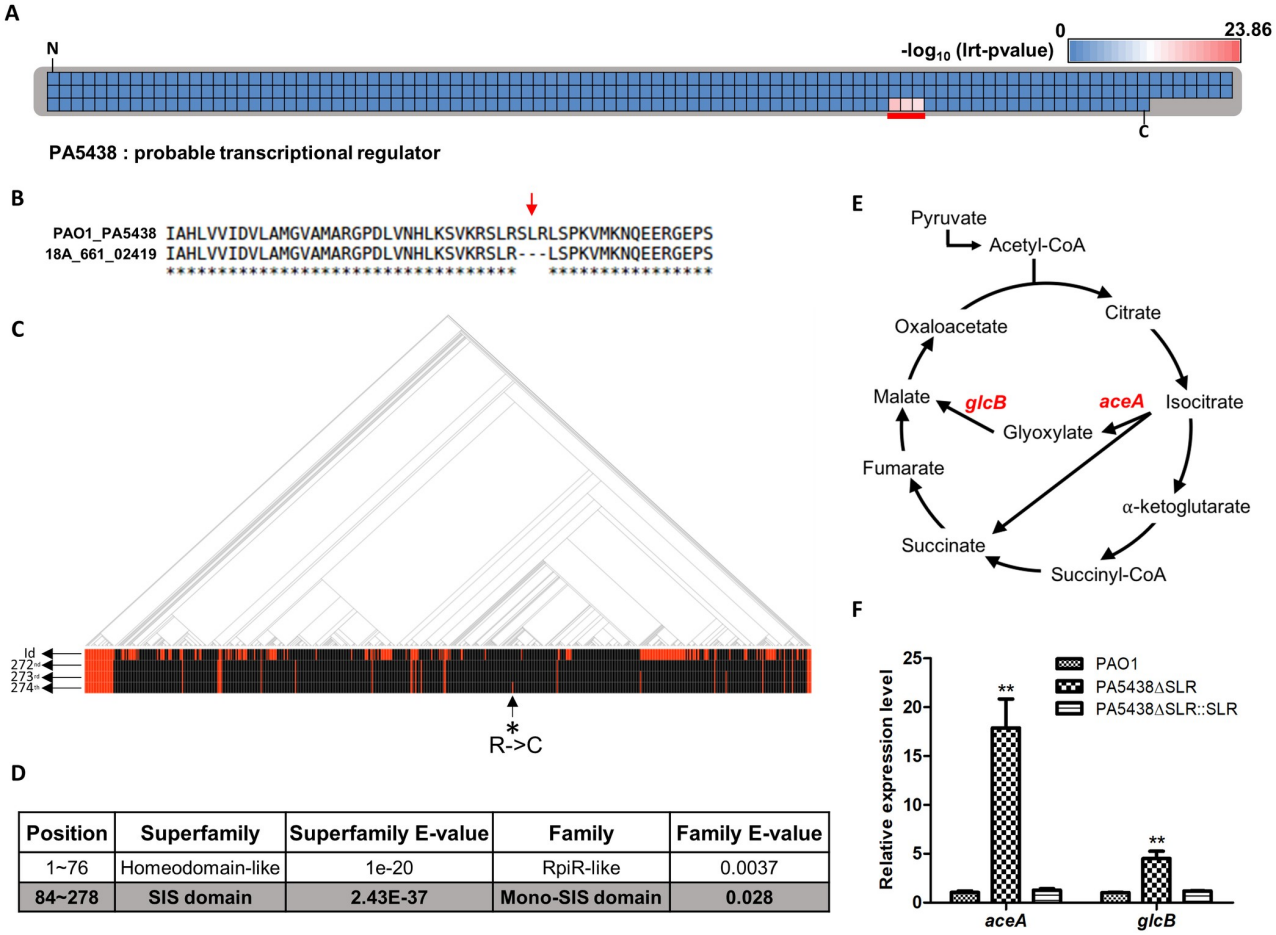
SLR deletion in PA5438 increases the expression level of *aceA* and *glcB*. **(A)** Total amino acid residues of PA5438 are presented and each amino acid locus is represented by individual squares. The color of each square indicates the lrt p-value assigned to that amino acid residue based on Pyseer results and colors are assigned according to the index above the heatmap, which was based on the negative logs of the maximum and minimum lrt p-values from the Pyseer result of amino acid locus. ‘N’ and ‘C’ mark the N- and C-terminus of PA5438, respectively. Residues at locus 272–274 of PA5438 with significant lrt p-values are underlined in red. **(B)** Comparison of the reference PA5438 to a homolog protein from a CF-isolated genome (18A_661_02419) is shown. The red arrow indicates deletion of SLR residues in PA5438 and corresponds to the red-underlined locus observed in **A**. **(C)** Phylogenetic tree of 636 genomes and their associated disease status are shown. Red and black lines in the ‘id’ row indicate genomes isolated from CF and non-CF patients, respectively. Red lines in the second, third, and fourth rows (272^nd^, 273^rd^, and 274^th^) each correspond to the absence of S, L, R residues, respectively. One exception to this is the red line in row 274 (asterisk-arrow), which was detected as R substituted by C. **(D)** A domain search result of PA5438 by SUPERFAMILY 2 is shown and the position of the SLR sequence inside the SIS domain is highlighted in bold. **(E)** AceA (isocitrate lyase) and GlcB (malate synthase) are enzymes involved in the glyoxylate shunt pathway (highlighted in red). **(F)** RNAs of PAO1, the PA5438ΔSLR mutant, and the complementation strain were extracted at OD_600nm_ ~1.0 in LB medium and relative expression levels of *aceA* and *glcB* were measured. **p<0.01.

Mutations at amino acid loci 272–274 (SLR) in a probable transcriptional regulator gene (PA5438) were strongly associated with CF isolates (Table 2 and Fig 3A). The amino acid sequence ‘SLR’ was deleted in the 2,419^th^ gene of the CF isolate 18A_661, a PA5438 homolog (Fig 3B). The SLR deletion in the PA5438 protein exhibits a highly positive correlation with CF isolates overall (Fig 3C), and the PA5438 sequences (except for the SLR sequence region) were exactly identical to the reference PA5438 sequence. Domain search with SUPERFAMILY 2 [41] predicted these amino acids to be a part of a sugar isomerase (SIS) domain (Fig 3D). Therefore, we constructed an in-frame deletion mutant, PA5438ΔSLR, to determine whether these three amino acids affect the transcriptional regulator function or not.

PA5438 is a transcriptional repressor that directly binds to the promoter region of the *aceA* (isocitrate lyase) gene and has been shown to repress the expression of the *glcB* (malate synthase) gene during growth in a non-C_2_ carbon source [42]. These repressed genes encoding enzymes involved in the glyoxylate shunt (GS) pathway (Fig 3E). To determine whether the suppressive activity of PA5438 is lost in the PA5438ΔSLR mutant, gene expression levels of *aceA* and *glcB* were measured by quantitative real-time PCR (qRT-PCR). The qRT-PCR results show that the expression level increased 17-fold for *aceA* and 5-fold for *glcB* (Fig 3F). Based on these findings, we expect that the deleted SLR sequence in PA5438 is an important region for determining *aceA* and *glcB* expression levels.

**Fig 4:**
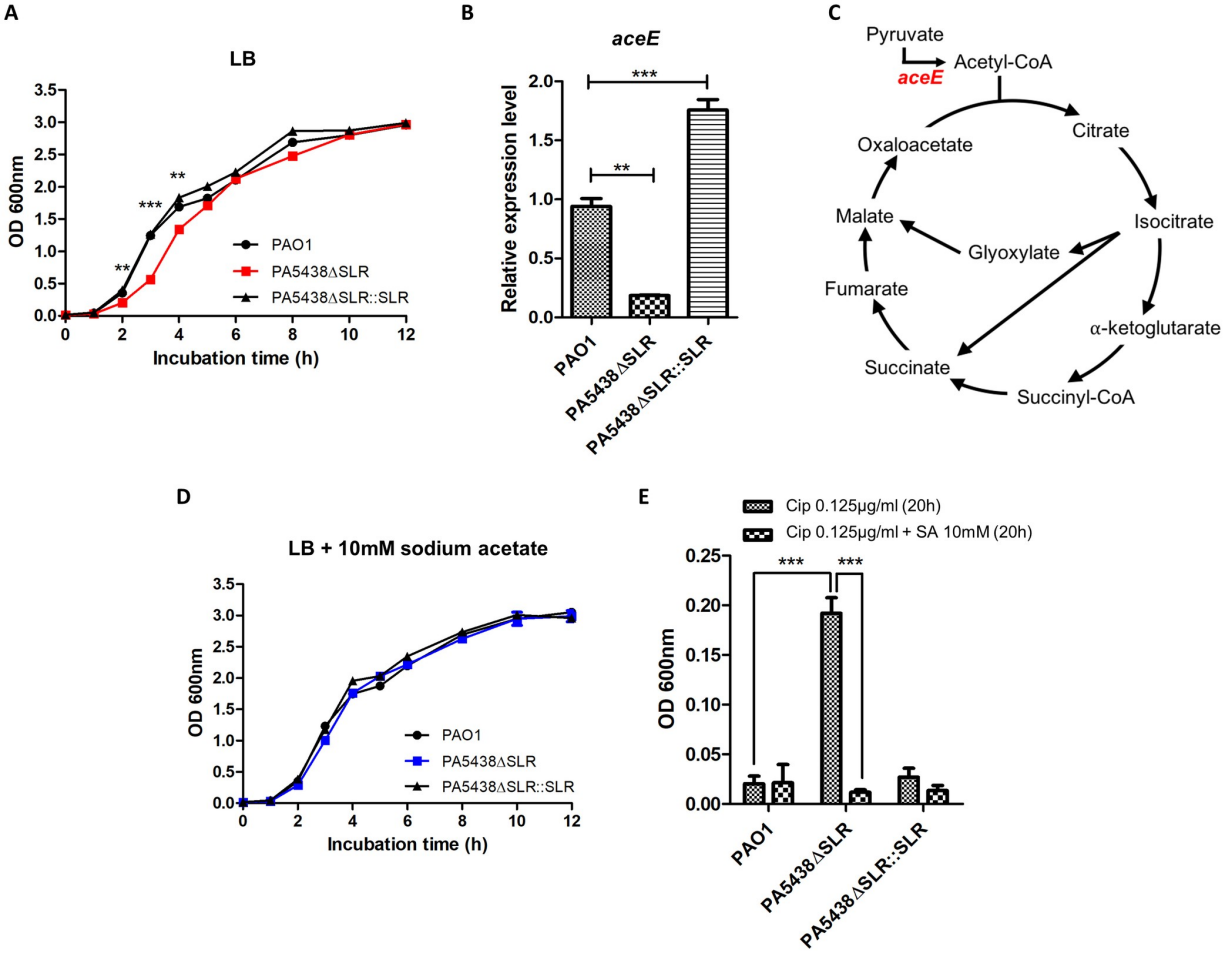
SLR deletion in PA5438 affects antibiotic susceptibility in LB medium. **(A)** Growth curves of PAO1, PA5438ΔSLR mutant, and the complementation strain in LB were observed over 12 hours. **p<0.01; ***p<0.001 **(B)** RNAs of PAO1, PA5438ΔSLR mutant, and the complementation strain were extracted at OD_600nm_ ~1.0 in LB and relative expression levels of *aceE* were measured. **p<0.01; ***p<0.001 **(C)** AceE (pyruvate dehydrogenase) is an enzyme involved in the entrance of The Krebs Cycle (highlighted in red). **(D)** Growth curves of PAO1, PA5438ΔSLR mutant, and the complementation strain in LB supplemented with 10mM sodium acetate were observed over 12 hours. **(E)** Antibiotic susceptibility tests against ciprofloxacin (Cip) and 10 mM sodium acetate (SA) were performed. Initial OD_600nm_ of PAO1, mutant, and the complementation strain were adjusted to 0.005, and OD_600nm_ was measured after 20 hours of static incubation in LB supplemented with Cip. The concentration of antibiotic was 0.125 μg/ml. ***p<0.001.

Further investigation of the PA5438ΔSLR mutant was performed to identify phenotypes that may aid in the adaptation of the strain to the CF environment. First, we assessed the phenotype of the mutant PA5438ΔSLR in LB media as growth rates of diverse CF isolates in LB media closely approximate those of CF isolates grown in artificial sputum medium (ASM) and synthetic CF sputum medium (SCFM) [17]. Slower growth in LB was observed in the PA5438ΔSLR mutant compared to PAO1 and the complementation strain, especially during the exponential phase (Fig 4A). In a recent study, RccR of *Pseudomonas fluorescens*, a PA5438 homolog, was shown to control pyruvate metabolism as well as the GS pathway [43]. Interestingly, pyruvate dehydrogenase (*aceE*) and *aceA* were regulated differently in the presence of 2-keto-3-deoxy-6-phosphogluconate (KDPG), which is bound to RccR [43]. We measured the relative expression level of *aceE* at the exponential phase because pyruvate metabolism affects growth. The expression of *aceE* in the mutant was suppressed compared to wildtype PAO1 and the complementation strain (Fig 4B). AceE generates acetyl-CoA from pyruvate, which feeds into the TCA cycle (Fig 4C). Therefore, we considered if the addition of metabolites related to this enzyme reaction (either as a precursor or product) would achieve recovery of growth. A defect in mutant growth was still observed with the addition of sodium pyruvate and sodium citrate, but the addition of sodium acetate as a substitute of acetyl-CoA resulted in a complete recovery of growth (Fig 4D).

Since antibiotic tolerance can be associated with slow-growing or non-dividing “persister” bacteria [44], we measured susceptibilities of PAO1, PA5438ΔSLR and the complementation strain to different classes of antibiotics (tobramycin and ciprofloxacin) commonly used to treat PA infection in CF patients. In static cultures with tobramycin or ciprofloxacin, the PA5438ΔSLR mutant reached a significantly higher OD_600nm_ in ciprofloxacin compared to wildtype PAO1 and the complementation strain, but only slightly higher OD_600nm_ in tobramycin (Figs 4E and S2A). Since growth in LB recovered with sodium acetate (Fig 4D), we performed antibiotic susceptibility tests with sodium acetate supplementation. A limited increase of tobramycin susceptibility was observed in PA5438ΔSLR (S2A Fig), whereas susceptibility to ciprofloxacin was fully restored to that of the wildtype PAO1 and complementation strain (Fig 4E). Therefore, we postulate that slower growth is involved in the decreased susceptibility of the PA5438ΔSLR mutant to the two antibiotics.

Evidently, the composition of nutrients in LB media is very different to that of the CF sputum. Therefore, a synthetic medium was prepared to mimic the nutritional makeup of CF sputum (SCFM) and used it to measure growth. Unlike growth in the LB medium, in SCFM, wildtype PAO1, PA5438ΔSLR and the complementation strain showed similar growth (S2B Fig). Since the decreased expression level of *aceE* seemed to be an important factor of growth in LB medium, we measured the expression levels of *aceE* in PA cultured in SCFM. The expression level of *aceE* in the PA5438ΔSLR mutant was similar to that of the wildtype PAO1 and complementation strain (S2C Fig). In contrast, *aceA* and *glcB* expression levels were significantly increased in the PA5438ΔSLR mutant compared to the wildtype and complementation strain grown in SCFM (S2D Fig).

**Fig 5:**
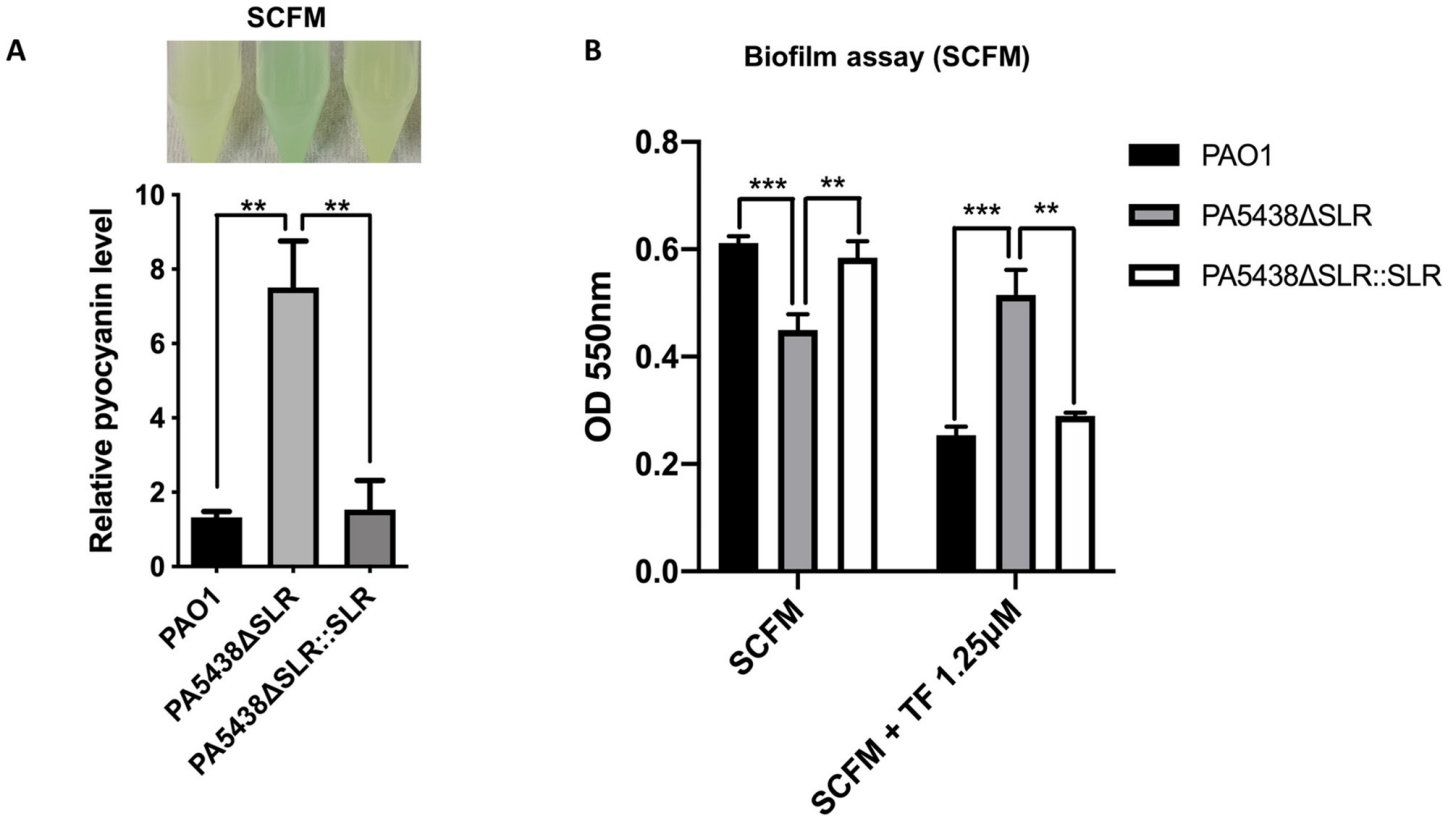
SLR deletion in PA5438 affects virulence and biofilm production in SCFM. **(A)** After bacterial culture of PAO1, PA5438ΔSLR mutant, and the complementation strain in SCFM for 8 hours with shaking, a pyocyanin assay was performed with culture supernatant, and relative pyocyanin level was calculated using PAO1 pyocyanin level. Bacterial cultures used in pyocyanin assay are shown. **p<0.01 **(B)** After 32 hours of static culture of PAO1, PA5438ΔSLR mutant and the complementation strain in SCFM and SCFM supplemented with holo-transferrin (TF), biofilm was measured spectrophotometrically. **p<0.01; ***p<0.001.

PA produces pyocyanin, an important virulence factor derived from phenazine and forms blue-green pigments. Pyocyanin induces reactive oxygen species (ROS) generation by transferring electrons to oxygen and increasing neutrophil apoptosis as a way of disrupting the host immune system [45]. As such, the supernatant color of a PA culture can function as a proxy for the virulent nature of the bacterium. Interestingly, we observed the 8 hour culture supernatant of PA5438ΔSLR in SCFM to be greener than that of the PAO1 and the complementation strain (Fig 5A). When we confirmed the relative pyocyanin levels of this bacterial culture, the PA5438ΔSLR mutant in SCFM resulted in 6~7 times more pyocyanin than PAO1 and the complementation strain (Fig 5A). Pyocyanin stimulates iron removal from transferrin in bacteria incubated under low oxygen conditions [46], and the level of transferrin is increased in the bronchoalveolar fluid from CF patients, compared to that of healthy individuals [47]. Therefore, we measured biofilms in which oxygen is limited, using the iron-saturated holo-transferrin. Compared to PAO1 and the complementation strain, the PA5438ΔSLR mutant produced less biofilm in SCFM, as demonstrated by a decreased OD_550nm_ absorbance value. However, in the presence of transferrin, the mutant produced a similar level of biofilm whereas the PAO1 and the complementation strain produced less biofilms than in SCFM (Fig 5B).

#### Potential implications in CF airway infections

These findings have potential implications in CF airway infections. An altered fatty acid profile has been associated with the airways of CF patients. For example, higher concentrations of palmitic acid and oleic acid have been detected in CF airway samples [48]. Fatty acid degradation upregulates the glyoxylate shunt (GS) pathway [49], and PA isolated from the CF lungs can induce the expression of genes involved in fatty acid metabolism and the GS pathway [50]. Furthermore, mucin is a major energy source in the CF lung environment in addition to fatty acids, and the GS pathway is important for mucin degradation and consumption [51]. Meta-transcriptomic analysis of several CF sputum samples in another study also found that *aceA* was upregulated and genes associated with glucose transporters and glycolysis were significantly downregulated [52]. Based on these previous studies, we assumed that the activation of the GS pathway may provide an advantage for PA proliferation in CF lungs.

Previous observations of GS pathway upregulation [50,52] are probably due to the presence of CF isolates that contain the abnormal PA5438 gene. In the PA5438ΔSLR mutant, *aceA* and *glcB* expression levels were increased not only in LB but also in SCFM. To investigate if the end products of the upregulated expression of *aceA* and *glcB* are functional, we examined the sequences of AceA and GlcB from genomes that harbor ΔSLR. Both promoters and proteins of AceA and GlcB were highly conserved (Table A, Table B in S1 Table). This indicates that the SLR deletion is the sole contributor to the transcriptional regulation of these two genes.

Glyoxylate shunt pathway is necessary for surviving phagocytosis and is upregulated in response to ROS [53,54]. We performed a gentamicin protection assay using our strains and mouse bone-marrow-derived neutrophils to measure how well the PA5438 mutant endures ROS-enriched and nutrient-poor phagocytic environment. We did not observe a significant difference in the CFUs of the PA5438 mutant strain compared to the wild type and the complementation strains in our experiment (S2E Fig).

As CF isolates have similar growth rates in LB and SCFM [17], we chose LB as the medium in which to investigate phenotypic differences between the wildtype and mutant PA. However, we observed a marked difference between the growth of the PA5438ΔSLR mutant cultured in LB and SCFM. In addition, the bacterial culture head a greener colour when SCFM was used (Fig 5A) than when LB was used. Given that PA5438 is involved in energy metabolism [43], we hypothesized that the mutant and wildtype phenotypes may vary depending on the type of medium used (LB or SCFM). Therefore, virulence phenotypes of the mutant, such as the increased pyocyanin levels observed in SCFM, are more likely to occur in the CF environment than the decreased antibiotic susceptibility observed in LB cultures. We also examined the genes involved in pyocyanin biosynthesis in the genomes that carry the PA5438ΔSLR mutation. Whereas several genomes were found to carry mutations in several of these genes when compared with the PAO1 sequences, other genomes possess a highly conserved gene set (S3 Fig). Based on this finding, we anticipate that virulent phenotypes such as that of the PA5438ΔSLR mutant are likely to manifest in the CF lung environment.

Despite differences in the nutrient compositions of LB and SCFM, the mutant phenotypes observed in LB cultures provide evidence that PA5438 acts as a transcriptional regulator. RccR of *Pseudomonas fluorescens*, a PA5438 homolog, has been shown to possess an SIS domain, the binding affinity of which is regulated by 2-keto-3-deoxy-6-phosphogluconate (KDPG) [43]. This KDPG-dependent moderation of PA5438 binding affinity is also dependent on the gene with which PA5438 binds. In the absence of KDPG, the binding affinity of PA5438 to *aceE* is increased, whereas its binding affinity to *aceA* is decreased [43]. A domain search using the SUPERFAMILY 2 database detected the SLR amino acid sequence of PA5438 to be inside the SIS domain (Fig 3D). The qRT-PCR results showed a decreased *aceE* gene expression level and an increased *aceA* gene expression level in LB (Figs 3F and 4B). Based on these findings, we propose that the SLR amino acid sequence may be a key region to which KDPG binds. In contrast to LB culture, in SCFM culture the *aceE* gene expression by the PA5438ΔSLR mutant is similar to that of wildtype PAO1 and the complementation strain. This observation may be attributed to the preferential use of amino acids over glucose in SCFM, which may lead to a decreased intracellular level of KDPG in the exponential phase of PA cultured in SCFM, unlike in LB. Further investigations are needed to conclusively determine whether KDPG indeed binds to the SLR region of PA5438.

Furthermore, elastase and pyocyanin are well-characterized virulence factors regulated by quorum sensing. We performed an elastase assay to test whether quorum sensing is related to the increased pyocyanin level [20]. Similar to the pyocyanin assay results, which showed a significant increase in pyocyanin in the PA5438ΔSLR mutant, relative elastase activity was slightly increased in the mutant compared to wildtype PAO1 and the complementation strain (S2F Fig). Therefore, we anticipate the increased pyocyanin level to be mediated by the quorum-sensing system. Pyocyanin stimulates iron removal from transferrin when PA is grown in low oxygen conditions [46], and increased levels of transferrin have been observed in CF patients [47]. Biofilm is a matrix of extracellular polymeric substances which protects the bacterial cells beneath, and oxygen supply is limited in developed biofilms [55]. Biofilm production of the PA5438ΔSLR mutant is decreased compared to PAO1 and the complementation strain in SCFM. In SCFM with transferrin, the mutant maintains the level of biofilm production, whereas PAO1 and the complementation strain produced less biofilms (Fig 5B). This implies that the mutant maintenance of biofilm production level in the presence of transferrin may confer greater fitness in the transferrin-enriched CF environment. Based on our experimental results, we speculate that this mutant may be problematic in the CF lung due to its increased virulence and biofilms.

### Mutations in the L-cystine transporter (YecS, PA0313)

#### Identification and experimental validation

**Fig 6:**
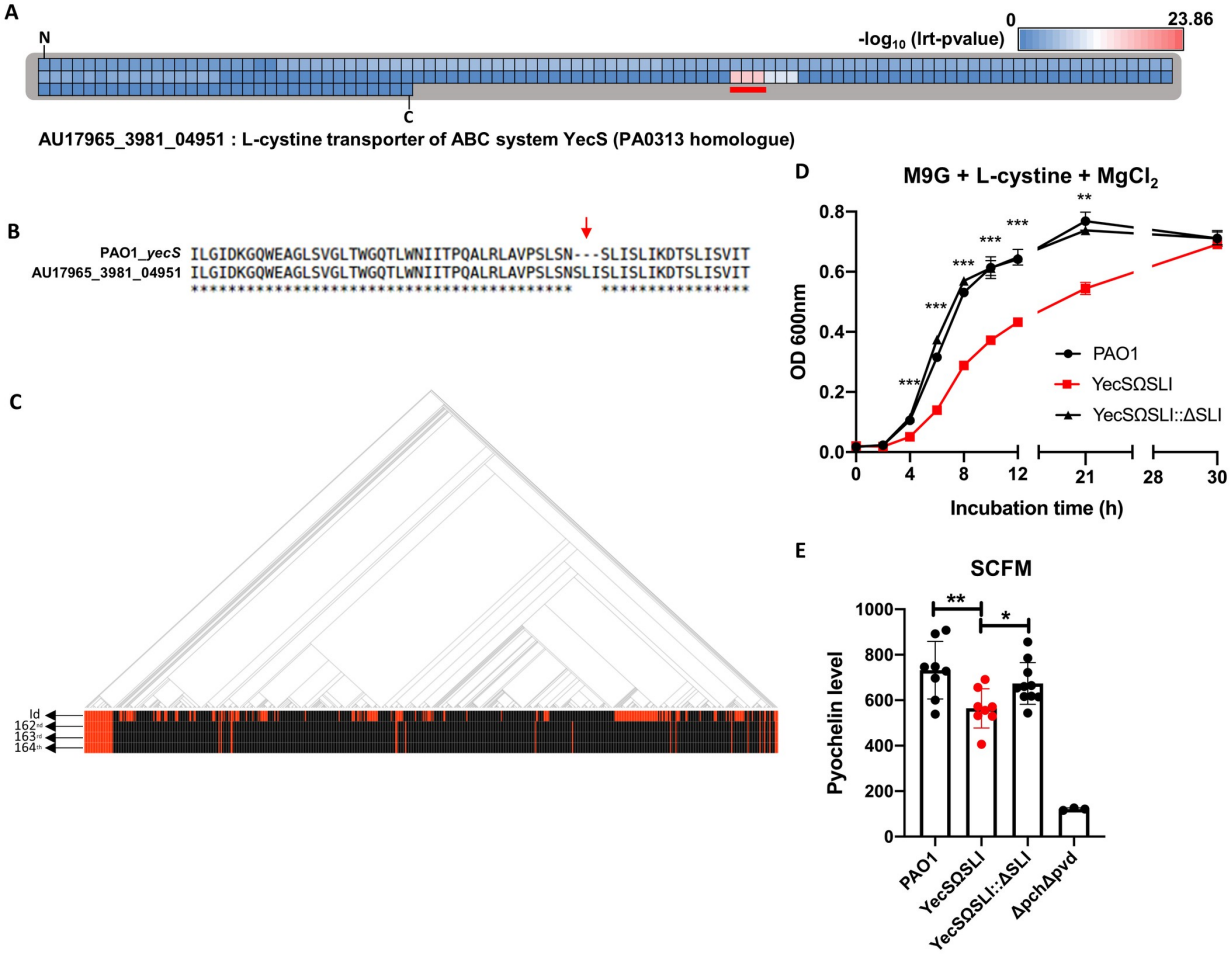
SLI insertion in YecS reduces L-cystine utilization activity. **(A)** The amino acid residues of CF-derived AU17965_3981_04951 protein are presented and each amino acid locus is represented by individual squares. The color of each square indicates the lrt p-value assigned to that amino acid residue based on Pyseer results and colors were assigned according to the index above the heatmap. The color index was generated based on the negative logs of the maximum and minimum lrt p-values from the Pyseer result of the amino acid locus. ‘N’ and ‘C’ mark the N- and C-terminus of AU17965_3981_04951, respectively. Residues presenting especially low lrt p-values are marked with a red underline and were found at loci 162–164 of the AU17965_3981_04951. **(B)** Comparison of the reference AU17965_3981_04951 to its PAO1 homolog YecS (PA0313) is shown. The red arrow indicates insertion of SLI residues in AU17965_3981_04951 and corresponds to the red-underlined locus observed in **(A)**. **(C)** Phylogenetic tree of 636 genomes and their associated disease status are shown. Red and black lines in the ‘id’ row indicate genomes isolated from CF and non-CF patients, respectively. Red lines in the second, third, and fourth rows indicate the presence of SLI insertion in PAO1 YecS. **(D)** Growth curves of PAO1, YecSΩSLI mutant and the complementation strain cultured in M9 minimal media with MgCl_2_ instead of MgSO_4_, supplemented with glucose and L-cystine as the sole sulfur source were measured over 30 hours. **p<0.01; ***p<0.001 **(E)** After bacterial culture of PAO1, YecSΩSLI mutant, the complementation strain and the Δ*pch*Δ*pvd* mutant, a pyochelin- and pyoverdine- deficient strain, in SCFM for 6 hours with shaking, the pyochelin in the bacterial culture supernatant was measured. *p<0.05; **p<0.01.

A major limitation of aligning 31-mers to the PAO1 reference genome is that the 31mers that do not align due to insertion mutations are overlooked. To determine the reference genes for such insertion mutations, un-aligned 31-mers were *de novo* assembled, and the contigs that formed were mapped to 635 genomes (excluding PAO1). The top 10 amino acid residues with corresponding 31-mers that do not align to the PAO1 genome are described in Table 3. Three amino acid residues at loci 162–164 (Fig 6) had significant lrt p-values compared to other amino acid residues. At these loci, the YecS homolog had insertions of three amino acid residues, SLI (Fig 6). Moreover, there was an additional copy of SLI in the AU17965_3981_04951 protein, comprising a total of three stretches of ‘SLI’, in contrast to a total of two stretches in the PAO1 YecS (S4 Fig). SLI insertions were strongly associated with CF isolates (Fig 6C) and the YecS homologs of these isolates were identical to the reference AU17965_3981 sequence, except for the additional SLI in PAO1 YecS. The AU17965_3981_04951 protein is a cytoplasmic-membrane transporter, and the additional ‘SLI’ sequence is thought to span both cytoplasmic and transmembrane regions (S5A and S5B Figs) [56].

To experimentally assess if the SLI insertion effects a phenotypic change, an in-frame mutant was constructed with the additional SLI sequence inserted into the corresponding locus of PAO1 YecS. No difference in growth was observed between the wildtype PAO1 and the YecSΩSLI mutant when both strains were grown in SCFM (S5C Fig). However, in M9 minimal media supplemented with glucose and L-cystine as the sole sulfur source, the mutant grew slower than PAO1 and the complementation strain, whereas growth in M9 media with an additional sulfur source present (MgSO_4_) showed no difference (Figs 6D and S5D).

Two L-cystine transporters are known in *E*. *coli*, an ATP-binding cassette (ABC) importer (FliY-YecSC) and symporter YdjN [57]. Previous work has shown that the presence of either of the two transporters is sufficient to meet the L-cystine requirement for normal growth when L-cystine is used as the sole sulfur source [57]. We performed a blastp search of 636 genomes using the database of 238 YdjN homologs [58] and concluded that 635 out of 636 genomes do not seem to encode a YdjN homolog (S5 Data). Therefore, based on our blast search and experimental result (Fig 6D), we hypothesize that PAO1 only possesses the ABC importer system for L-cystine uptake. Moreover, the decreased growth of the SLI insertion mutant is attributed to the decreased activity of the ABC transporter rather than a complete loss of function. A comparison of growth between a YecS clean deletion mutant and our SLI insertion mutant would help in better understanding the assortment of L-cystine transporters present in PA.

### Potential implications in CF airway infections

In addition to the SLI insertion mutation, we focused on other patterns of mutation in YecS that were observed across the CF isolate genomes. One such pattern is the deletion of an SLI sequence that results in CF isolates encoding just one SLI sequence in YecS homologs (S4A Fig). Another pattern of mutation observed by multiple alignments of YecS homologs is the deletion of long stretches of amino acid residues (S4B Fig). Since the SLI insertion mutant exhibited decreased L-cystine transporter activity (Fig 6D), we anticipate that such large deletions in the YecS protein would incur a complete loss of function.

Amino acid concentrations in the sputum vary across individual CF patients [59] and cysteine levels can be low or even undetectable [59]. Since cysteine is oxidized to cystine, a low level of one may indicate a low level of the other in the CF lung. Therefore, on one hand, large deletions in YecS may provide a survival advantage by allowing the CF isolate to avoid the energy cost of maintaining a functional YecS transporter in the cysteine-deficient CF lung environment. On the other hand, the reduction of intracellular cystine to cysteine contributes to ROS production in *E*. *coli* [60]. Thus, we expect small-scale mutations in YecS may be a result of evolution that protects the CF isolates from ROS stress in the cysteine-present CF lung environment. Additionally, pyochelin is a well-known siderophore produced by PA. Pyochelin may inflict tissue damage in the CF lung by inducing a continuous inflammatory response [61]. Interestingly, cysteine is a precursor molecule required for the production of pyochelin [62]. Therefore, we tested the effect of SLI insertion in YecS on pyochelin production of PAO1, the YecSΩSLI mutant, the complementation strain, and a Δ*pch*Δ*pvd* mutant [63] that does not produce pyochelin and pyoverdine. The SLI insertion mutant exhibited decreased pyochelin production compared to the wildtype and the complementation strain (Fig 6E). A decreased level of pyochelin may be associated with a less virulent phenotype of the mutant, which may be an adaptation strategy for establishing a chronic infection in the CF lung environment. Whereas several genomes carried mutations in pyochelin biosynthesis genes, when compared with the PAO1 sequences, other genomes possess a highly conserved gene set (S6 Fig). Therefore, it may be the case that less virulent phenotypes such as that of the YecSΩSLI mutant proliferate in the CF lung environment.

### Small-scale alterations in intergenic regions: phuS and phuR

**Fig 7:**
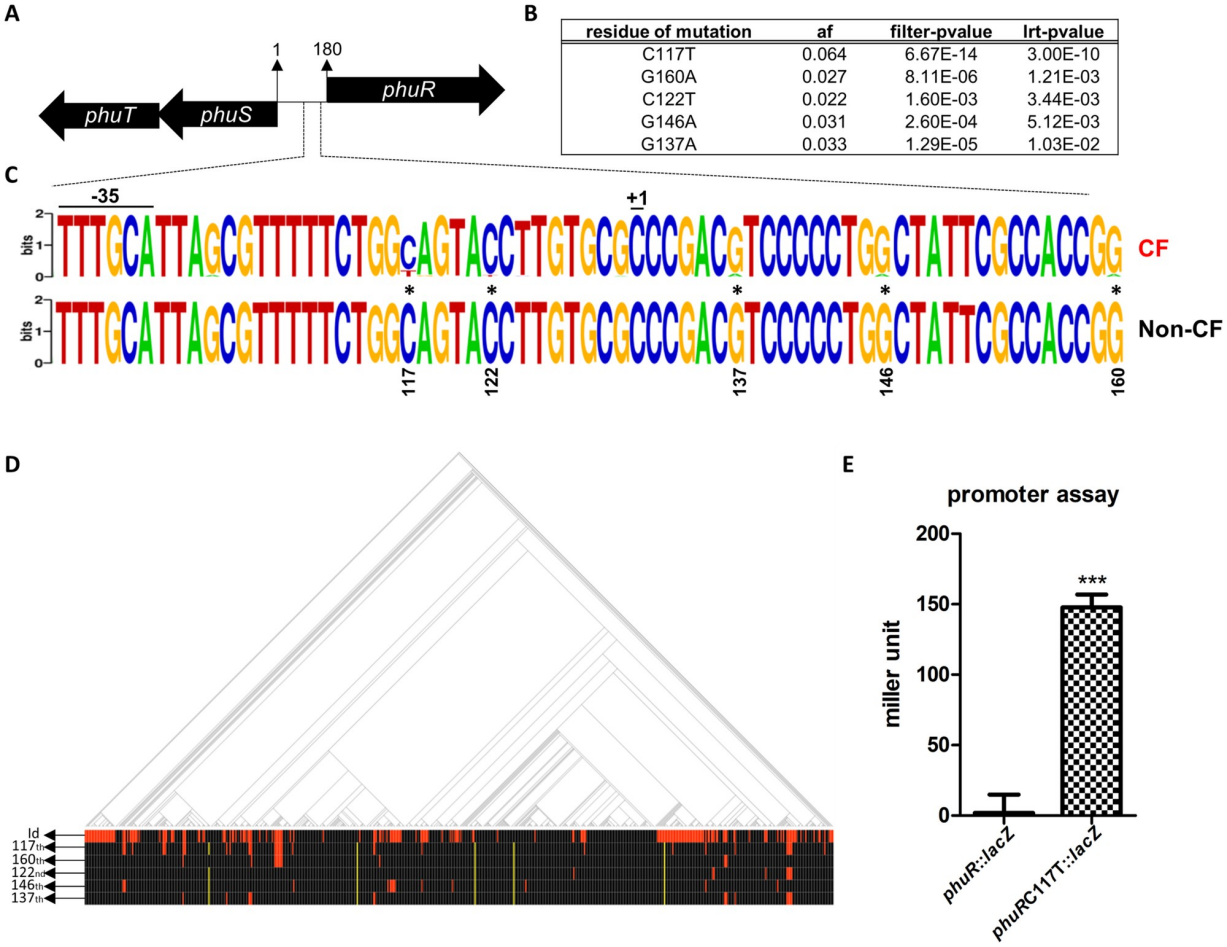
One SNP in the *phuR* promoter region increases expression level. **(A)** The 180 bp intergenic region between *phuR* and *phuS* is shown. **(B)** The top 5 associated SNPs detected using Pyseer within this intergenic region are listed and the number in the ‘residue of mutation’ column indicates the forward strand-based locus of mutation. The nucleotide in front of the locus number indicates the reference base in the intergenic region, whereas the nucleotide following the locus number indicates the changed base. Information contained in the following three columns is similar to those described in Fig 2B. **(C)** Promoter region of *phuR* (region from -35 to +1) based on the PRODORIC database [84] is visualized with WebLogo [85], and SNPs listed in **(B)** are marked by asterisks. The height of a stack reflects the degree of sequence conservation at that position, while the height of symbols within a stack indicates the relative frequency of each nucleotide at that position. The upper sequence is derived from the intergenic regions of CF genomes and the sequence below is based on intergenic regions of non-CF genomes. **(D)** Red and black lines in the ‘id’ row indicate genomes of CF and non-CF isolates, respectively. Red lines in the bottom five rows indicate mutations at each locus, shown in **(B)**, and yellow lines indicate that this intergenic region is missing from the genome. **(E)** Culture of *phuR*::*lacZ* and *phuRC117T*::*lacZ* grown to OD_600nm_ ~0.25 were used in a β-galactosidase assay, to compare *phuR* promoter activity. ***p<0.001.

Comparison of genomes using k-mers enables the investigation of intergenic regions. Of all contigs assembled from significantly different 31-mers, between CF and non-CF isolates, 29 contigs aligned to the non-coding regions of the PAO1 genome. Most aligned to either the hs region or rDNA sequences (Fig 1B). Since mutations located in the hs region may cause gene deletions, and rDNA sequence regions were excluded from analysis due to difficulties in interpretation, we selected regions other than these loci. Among several such intergenic regions, one intergenic region between *phuR* and *phuS* operons, was involved in the pseudomonas heme utilization (phu) systems (Fig 7A). The function of such a system is the acquisition of iron from the heme group of hemoglobin [64]. To evaluate the potential role of these mutations in CF adaptation, Pyseer analysis was performed using the nucleotide sequences of this intergenic region from PAO1 and other isolates (Fig 7B). The most significant hit was the transition of the 117^th^ residue on the forward strand from cytosine (C) to thymine (T) (Fig 7B). Two mutations (C117T and C122T) are included in the *phuR* promoter region (from -35 region to transcriptional initiation site (+1)) (Fig 7C).

Consistent with our findings, the frequency of mutations within this intergenic region in a CF isolate were found to increase significantly compared to the background mutation rate, and the mutated intergenic region increased *phuR* promoter activity [65]. In some cases, the intergenic region was deleted (Fig 7D) and the promoter activity is probably absent in these isolates. When such cases of promoter deletion are excluded from the analysis, a stronger correlation is expected between the mutations of this region and CF isolates. To examine whether the C117T point mutation affects the expression of *phuR*, we performed a promoter activity assay by measuring β-galactosidase activity. Promoters with this mutation exhibited increased activity (Fig 7E). Therefore, increased *phuR* promoter activity will ultimately result in increased phu system activity. We speculate that the C117T mutation is an important strategy for iron uptake and thus survival in the CF environment.

## Conclusions

**Fig 8:**
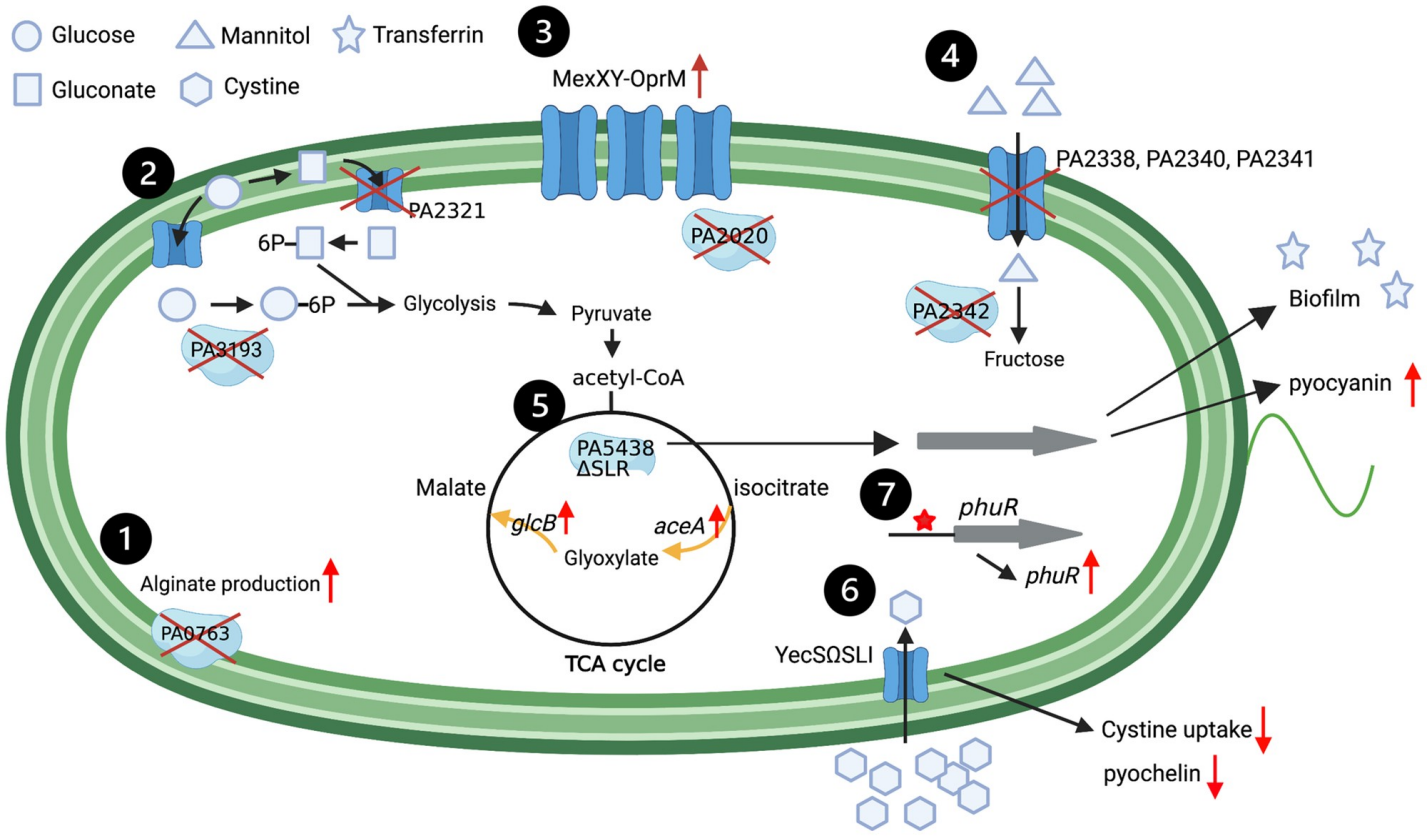
Schematic diagram with candidate genes. Gene products of both (1–4) large- and (5–7) small-scale mutations significantly found in CF are illustrated with BioRender.com. We anticipate several phenotypic changes to arise as result of these mutations: (1) Large-scale mutations in the MucA protein stimulate alginate production which is an important component of biofilm; (2) PA2321 and PA3193 are involved in glucose catabolism. Disruption of PA2321 and PA3193 may be results of evolution in the CF environment which is enriched in free amino acids, nutrients preferred by *P*. *aeruginosa*; (3) Large-scale mutations in the PA2020 regulator upregulates expression level of MexXY, which may in turn result in increased antibiotic resistance; (4) Mutations in mannitol utilization gene cassette may aid in defence against the combined treatment of aminoglycoside and mannitol; (5) SLR deletion in PA5438 induces the glyoxylate shunt pathway, pyocyanin production and maintenance of biofilm in the presence of transferrin; (6) SLI insertion in YecS decreases cystine uptake and pyochelin production; (7) Mutation in *phuR* promoter region increases *phuR* expression.

In this study, we compared a large set of genomes from clinical PA isolates (CF vs. non-CF) and identified mutations that occurred differentially in either CF or non-CF isolates using GWAS based on 31-mer counting (Fig 8). To integrate these findings within a functional genomics context [66], the impact on bacterial growth- and virulence-associated phenotypes was also assessed. Importantly, PAO1-derived variants, PA5438ΔSLR and YecSΩSLI, exhibited distinct phenotypes in *in vitro* assays. Moreover, a single nucleotide replacement in the promoter region of the *phuR* gene caused a considerable increase in gene transcription. Together, these results suggest that PA may benefit from small-scale mutations when establishing chronic infections in the CF airway. Mutations in the *lasR* gene encoding a QS regulator have been reported as frequent in CF isolates [7,8]. However, we found no evidence that mutations in LasR or other QS-related proteins were significantly overrepresented in CF or non-CF isolates. Given that the MucA mutation was represented in our analysis, demonstrating the precision of our bioinformatic approach, the *lasR* mutation might in fact be a common feature in PA, either causing chronic CF infection or other acute infections. Finally, while our study improves understanding the genetic changes in PA associated with chronic CF airway infection, they do not reveal the order in which these genetic changes occurred. Evaluating the chronology of adaptative changes in the CF lung will be important for explaining the persistence of PA.

### Experimental procedures

#### Bioinformatic analysis

Overall, 2,187 PA genomes were downloaded from the Pseudomonas Genome Database [27], and 1,001 genomes with information on the host disease were selected. A phylogenetic tree was created with RapidNJ, using Snippy-generated alignments of the core genome as input [67,68], and visualized with Microreact [69]. Based on the initial tree, the selection of genomes was trimmed down with Treemmer [70] to include 636 isolates while maintaining 99.8% of the original diversity (S1 Data). The prediction of protein-coding genes in the 636 genomes was performed using Prokka [31]. K-mers of 31 base pairs length (31-mers) were counted in the 636 individual genomes with Fsm-lite [71]. A similarity matrix was constructed with Snp-sim [72] using a core alignment of 636 genomes generated by Snippy [68]. Pyseer [29], which uses a mixed model (FaST-LMM), was run using the counted k-mers and the similarity matrix as input, and lrt p-values were assigned to each 31-mer. To determine the 31-mers that were significantly different between CF and non-CF groups, 31-mers with poor chi values (indicative of invalid 𝜒^2^ test) or lrt p-values above 2.8e-08 (cutoff value in this analysis) were removed. As a result, 41,685 31-mers were detected to have significant associations with either disease status (CF or non-CF). Distribution of these 31-mers aligned to the PAO1 whole genome was visualized with Phandango [30]. Subsequently, *de novo* assembly of these 31-mers was completed using Trinity [73]. Overall, 489 contigs were constructed, and these contigs mapped to 29 intergenic regions, six untranslated regions, and 109 translated genes of the PAO1 genome with Blastn. Individual contigs that aligned to either protein-coding regions or intergenic regions were blasted against 635 genomes (the PAO1 genome was excluded) [74]. The top hit from each pairwise alignment with a blast e-value <0.01 was chosen as a homolog of the candidate sequence in each isolate.

In 453 contigs mapping to protein-coding regions, homologs were clustered under several conditions of similarity and gene length coverage (S95L95 -: 95% similarity and 95% length coverage; S70L95–70% similarity and 95% length coverage) using Blastclust [74]. Homologs that clustered with the reference PAO1 protein were assigned as ‘1’ and those that did not were assigned ‘0’. We then used the clustering matrix as input for Pyseer. Clusters with either poor chi values or lrt p-values larger than 3.22E-05 were removed. In this manner, genes with multiple co-localized mutations were determined and defined as ‘large-scale’ mutations (Fig 1C). Other protein-coding region mutations were treated as ‘small-scale’ mutations.

Where contigs mapped to protein-coding regions or intergenic regions, multiple alignment of the candidate sequence and its homologs was performed using Mafft [75]. This allowed characterization of nucleotides (for intergenic region candidates) or amino acid sequences (for protein-coding genes) at each locus for detecting small-scale mutations. If the residue of a homolog was identical to that of the reference PAO1 intergenic region or protein, it was assigned a value of ‘1’ at that locus, and if not, a value of ‘0’ was assigned. In this way, a locus matrix was created for use as the input for Pyseer and loci with either poor chi values or lrt p-values larger than 4.01E-06 were removed.

Finally, k-mers that failed to align to the PAO1 genome were also analyzed. However, the PAO1 reference gene in these cases was replaced with the reference gene detected in alternative isolate genomes. As such, we were able to investigate the presence of insertion mutations. For KEGG functional annotation and pathway analysis, BlastKoala [34] was used with large-scale mutations. Sequence comparison was performed by Clustalw [76] and multiple alignment was visualized with Jalview [77]. SUPERFAMILY 2 [41] search was used to investigate the domains of the PA5438 gene. Transmembrane domain prediction of the AU17965_3981_04951 protein was carried out using Phobius [56].

#### Bacterial strains and growth conditions

All bacterial strains and plasmids used in this study are shown in Table C in S1 Table. PAO1 was used as a reference strain [1]. Bacterial cultures were grown in LB medium (1% [w/v] tryptone, 0.5% [w/v] yeast extract, and 1% [w/v] sodium chloride) at 37°C and Synthetic Cystic Fibrosis Medium (SCFM) using a previously described protocol [59]. Single bacterial colonies were picked from LB plates and inoculated in fresh LB or SCFM broths for precultures and grown overnight. Precultures were diluted 100-fold in fresh LB broth to subculture and incubated at 37°C with shaking at 230 rpm. For SCFM cultures, bacterial preculture was diluted and inoculated to the starting OD_600nm_ 0.025 in fresh SCFM broth for subculture. All SCFM cultures were incubated under the same conditions as LB cultures, except for the incubation time, which was dependent on specific experimental procedures.

### In-frame mutant construction

All in-frame mutants, including an SLI insertion in the YecS protein and SLR deletion in the PA5438 protein, were constructed from the PAO1 strain. In-frame insertions and deletions were performed to construct mutants with amino acid level changes. In the case of the in-frame deletions, the 5′ and 3′ flanking regions were designed to overlap in both directions of SLR. For the in-frame insertion, nucleotide sequences corresponding to amino acid sequence ‘SLI’ were inserted into the middle of the 5′ flanking region. An overlap was constructed by using each 5′ primer of 5′ flanking region and 3′ primer of 3′ flanking region. For both deletion and insertion mutations, an overlapping product was inserted into modified pCVD442, a suicide vector, containing gentamicin and ampicillin resistance markers. PAO1, grown on LB agar, was conjugated with *E*. *coli* SM10 λpir, harboring pCVD442 with the overlapping product inserted, grown on LB agar with 50 μg/ml ampicillin and 30 μg/ml gentamicin. Conjugates were spread onto LB agar with 50 μg/ml gentamicin and 20 μg/ml irgasan to select single crossover recombinants. The single crossover recombinant was incubated on LB agar without NaCl but containing 8% sucrose for the selection of the desired mutant. Sequence verification was performed by PCR. Primers used in constructing these mutants are listed in Table D in S1 Table.

### Complementation

Identical primers were used in constructing the aforementioned mutants (Table D in S1 Table; PA5438#1, PA5438#4, yecS#1, and yecS#6), and were used to amplify the *yecS* and *PA5438* sequences of wildtype PAO1. The amplified wildtype sequences were each inserted into pCVD442, and identical steps of mutant construction were followed to generate the complementation strains of the YecSΩSLI and PA5438ΔSLR mutants.

### Promoter assay

To perform the *phuR* gene promoter assay, intergenic regions (180 bp) between *phuS* and *phuR* in the PAO1 genome and AU2342_3932 [78] with only the 117^th^ locus changed from cytosine to thymine were amplified with primers listed in Table D in S1 Table. These PCR-products were each cloned into the upstream region of the β-galactosidase gene of puc18-mini-Tn7t-Gm-LacZ [79] for chromosomal insertion. The constructed plasmid was transformed into *E*. *coli* DH5α λpir. After verification by DNA sequencing, the plasmids with the helper plasmid pTNS2 that encodes TnsABC+D genes (allowing Tn7 transposition) were electroporated into PAO1. Empty puc18-mini-Tn7t-Gm-LacZ plasmid with no insert was used as a control to measure the baseline expression of *lacZ*. The potential clones were selected on LB agar with 50 μg/ml gentamicin and sequence verification was performed to select the final candidates whose transposon was inserted properly into the region following the *glmS* gene. β -galactosidase activities of the three clones (con::*lacZ*, *phuR*::*lacZ*, and *phuR*C117T::*lacZ*), summarized in Table C in S1 Table, were grown in LB and measured at the exponential phase (OD_600nm_ ~0.25).

### Growth curves

Wildtype PAO1, the PA5438ΔSLR mutant, and the complementation strain were precultured overnight in LB broth. Precultures were diluted 100-fold in fresh LB and incubated at 37°C with shaking at 230 rpm. Growth in LB was observed over 12 hours and the OD_600nm_ was measured. Growth in SCFM was detected in the same manner over a period of 8 hours. Growth recovery tests of the PA5438ΔSLR mutant were performed using LB medium and LB supplemented with 10 mM sodium acetate (Sigma-Aldrich, USA), 10 mM sodium pyruvate (Sigma-Aldrich, USA), and 10 mM sodium citrate (Sigma-Aldrich, USA), and OD_600nm_ were measured at regular time intervals. To examine the functional capacity of the L-cystine transporter, overnight precultures of PAO1, the YecSΩSLI mutant, and the complementation strain were washed in phosphate-buffered saline (PBS), and diluted to OD_600nm_ ~ 0.025 in fresh M9 minimal media supplemented with 22.2 mM glucose and L-cystine (0.25 mM) as the sole sulfur source (2 mM MgCl_2_ instead of MgSO_4_). OD_600nm_ was measured over 30 hours. Growth was also measured over 21 hours under identical conditions, except for the inclusion of 2 mM MgSO_4_ (instead of MgCl_2_) in M9 minimal media.

### Antibiotic susceptibility test

For antibiotic susceptibility tests, ciprofloxacin (Duchefa, The Netherlands) and tobramycin (Sigma-Aldrich, USA) were used. Wildtype PAO1, the PA5438ΔSLR mutant, and the complementation strain were precultured overnight in LB broth. Bacterial preculture was diluted in LB containing the relevant antibiotics (ciprofloxacin or tobramycin) and the starting OD_600nm_ was adjusted to 0.005. For static cultures, the antibiotic concentrations used were 0.125 μg/ml ciprofloxacin and 1 μg/ml tobramycin. OD_600nm_ was measured after 20 and 26 hours of static incubation with ciprofloxacin and tobramycin, respectively. An antibiotic susceptibility test with 10 mM sodium acetate supplementation was conducted under the same conditions.

### Reverse transcription and quantitative real-time PCR

PAO1, the PA5438ΔSLR mutant, and the complementation strain were precultured and subcultured in LB. After incubating the subcultures to OD_600nm_ ~1.0, RNeasy Mini kit (Qiagen, Netherland) and on-column DNase1 digestion were used to extract RNA following the manufacturer’s protocol. 1 μg of RNA was reverse-transcribed to synthesize complementary DNA by using reverse transcriptase (Takara Bio, Japan) and random hexamer primers. To check for DNA contamination, the same cDNA synthesis was performed in the absence of reverse transcriptase. SYBR green-based qPCR was performed using an ABI 48-well StepOne real-time system. The primers used are listed in Table D in S1 Table. CT values were normalized by 16S rRNA CT values. SCFM cultures (OD_600nm_ ~0.9) followed identical RNA extraction and qRT-PCR procedures.

### Elastase and pyocyanin tests

Precultures of PAO1, the PA5438ΔSLR mutant, and the complementation strain were prepared for elastase and pyocyanin tests in SCFM as described above. Bacterial supernatant was harvested from bacterial subcultures incubated for 8 hours in SCFM, and assays were performed as described previously [80,81]. Elastase and pyocyanin values were normalized using OD_600nm_.

### Pyochelin measurement

Precultures of PAO1, the YecSΩSLI mutant, and the complementation strain in SCFM were prepared as described above. Bacterial supernatant was harvested from bacterial subcultures, incubated for 6 hours in SCFM, and fluorescence was measured using Varioskan Flash 3001 (Thermo Scientific, USA) at excitation wavelength 355 nm and emission wavelength 440 nm [82]. Pyochelin values were normalized using OD_600nm_.

### Biofilm assay

Precultures of PAO1, PA5438ΔSLR mutant, and the complementation strain in SCFM were prepared as described above. Bacterial precultures were diluted in SCFM or SCFM containing the holo-transferrin (Sigma-Aldrich, USA) in a 96-well plate, and the starting OD_600nm_ was adjusted to 0.025. For static cultures, the holo-transferrin concentration used was 1.25 μM. After 32 hours of static incubation, biofilm assay was performed as described previously [83].

### In vitro gentamicin protection assay

Neutrophils were isolated from the bone-marrows of C57BL/6 mice following the protocol of EasySep Mouse Neutrophil Enrichment Kit (STEMCELL, Canada). Following isolation, neutrophils were maintained in Opti-MEM media (ThermoFisher Scientific, USA). Precultures of PAO1, the PA5438ΔSLR mutant, and the complementation strain in SCFM were prepared in the manner described above. Bacterial culture was harvested from bacterial subcultures incubated for 4 hours in SCFM. The initial infection dosage was 10^7^ CFU per 5 ✕ 10^5^ neutrophils. After 2 hours of co-culturing *P*. *aeruginosa* and neutrophils, the culture supernatant was removed and fresh Opti-MEM supplemented with 50 μg/ml gentamicin was added for 1 hour. After performing wash twice in PBS, 0.5% Triton-X was added and bacteria number were counted by using pseudomonas isolation agar (Sigma-Aldrich, USA).

### Statistical analysis

Data are expressed as mean ± standard deviation. Unpaired Student’s t-test (one-tailed, unequal variance) was performed to analyze the differences between experimental groups. P-values smaller than 0.05 were considered statistically significant. All experiments were repeated for reproducibility.

## Supporting information

S1 Fig Phylogenetic tree constructed with CF and non-CF genomes. Upper phylogenetic tree was constructed with 1,001 genomes containing host disease information, and the tree below was drawn using 636 genomes and maintaining 99.8% diversity of the upper tree. Black and red leaves each indicate non-CF and CF isolates, and the numbers of CF and non-CF genomes for constructing each phylogenetic tree are placed inside brackets.
(TIF)

S2 Fig Phenotypes of the PA5438ΔSLR mutant. **(A)** Antibiotic susceptibility test with tobramycin (Tob) and Tob with 10 mM sodium acetate (SA) were performed. Initial OD_600nm_ of PAO1, PA5438ΔSLR mutant, and the complementation strain were adjusted to 0.005, and OD_600nm_ was measured after 26 hours of static incubation in LB supplemented with Tob. The concentration of antibiotic was 1 μg/ml. **p<0.01 **(B)** Growth curves of PAO1 and PA5438ΔSLR mutant in SCFM were observed over 8 hours. **(C)** RNAs of PAO1, the PA5438ΔSLR mutant, and the complementation strain were extracted at OD_600nm_ ~0.9 in SCFM and relative expression levels of *aceE* were measured. **(D)** Relative expression levels of *aceA* and *glcB* of the same RNA used in **(C)** were measured. ***p<0.001 **(E)** The initial infection dosage was 10^7^ CFU per 5 ✕ 10^5^ neutrophils isolated from the bone-marrows of C57BL/6 mice. After 2 hours of co-culturing *P*. *aeruginosa* and neutrophils, we added gentamicin to the culture medium for 1 hour to remove any extracellular bacteria. We then harvested the intracellular bacteria by treating the neutrophils with 0.5% Triton-X, and measured the bacterial CFU. **(F)** After bacterial culture of PAO1, PA5438ΔSLR mutant, and the complementation strain in SCFM for 8 hours with shaking, an elastase assay was performed with culture supernatant. **p<0.01.
(TIF)

S3 Fig Blastp results of genes associated with pyocyanin biosynthesis. Each row represents a genome containing an SLR deletion in PA5438 homologs and color indicates whether it is included in CF or non-CF. Columns contain PAO1 proteins associated with pyocyanin biosynthesis and similarity (S), % of identical matches between reference PAO1 protein and its homolog, and length coverage (L), % of reference PAO1 protein sequence covered by its homolog. Gray box indicates there is no homolog under e-value 0.01 in the blastp search.
(TIF)

S4 Fig Multiple alignment of YecS and its homologs. Multiple alignment of YecS and its homologs is visually represented. Names of the genomes are shown to the left of the multiple alignment. PAO1 and AU17965_3981 are representative genomes of non-CF and CF groups, and genomes labeled with red or blue belong to the CF or non-CF groups, respectively. Black arrow marks where the additional SLI insertion occurs (162^nd^ to 164^th^ residues) compared to the YecS protein. Regions marked by asterisks are regions of SLI amino acid repeat sequences in YecS. Genomes in **a** contain a deletion of SLI, resulting in a single copy of SLI, and genomes in **b** present large deletions in YecS.
(TIF)

S5 Fig Transmembrane domain prediction and growth curves of the YecS mutant. **(A)** Predicted transmembrane domains of the AU17965_3981_04951 protein are portrayed. Numbers below the figure indicate the amino acid loci. **(B)** Detailed amino acid ranges of the predicted transmembrane domains are listed. The SLI insertion in the AU17965_3981_4951 protein is present within the region highlighted in bold. **(C)** Growth curves of PAO1 and YecSΩSLI mutant in SCFM were measured over 8 hours. **(D)** Growth curves of PAO1 and YecSΩSLI in M9 minimal media supplemented with glucose, L-cystine, and MgSO_4_ were recorded over 21 hours.
(TIF)

S6 Fig Blastp results of genes associated with pyochelin biosynthesis. Each row represents a genome containing an SLI insertion in YecS homologs and color indicates whether it is included in CF or non-CF. Columns contain PAO1 proteins associated with pyochelin biosynthesis and similarity (S), % of identical matches between reference PAO1 protein and its homolog, and length coverage (L), % of reference PAO1 protein sequence covered by its homolog. Gray box indicates there is no homolog under e-value 0.01 in the blastp search.
(TIF)

S1 Data Information of 636 genomes. (XLSX)

S2 Data Pyseer results. (XLSX)

S3 Data Cluster and locus information of 636 genomes. (XLSX)

S4 Data Amino acid sequences of small insertion variants from isolates other than PAO1. (DOCX)

S5 Data Blastp results with 636 genomes and 238 YdjN homologs. (XLSX)

S1 Table Supplementary tables. (DOCX)
