# Supplementary figures and images for: Genome-wide association study of signature genetic alterations among pseudomonas aeruginosa cystic fibrosis isolates
Source: PLoS Pathog. 2021 Jun 23;17(6):e1009681. doi: 10.1371/journal.ppat.1009681 (PMC8274868; doi:10.1371/journal.ppat.1009681)

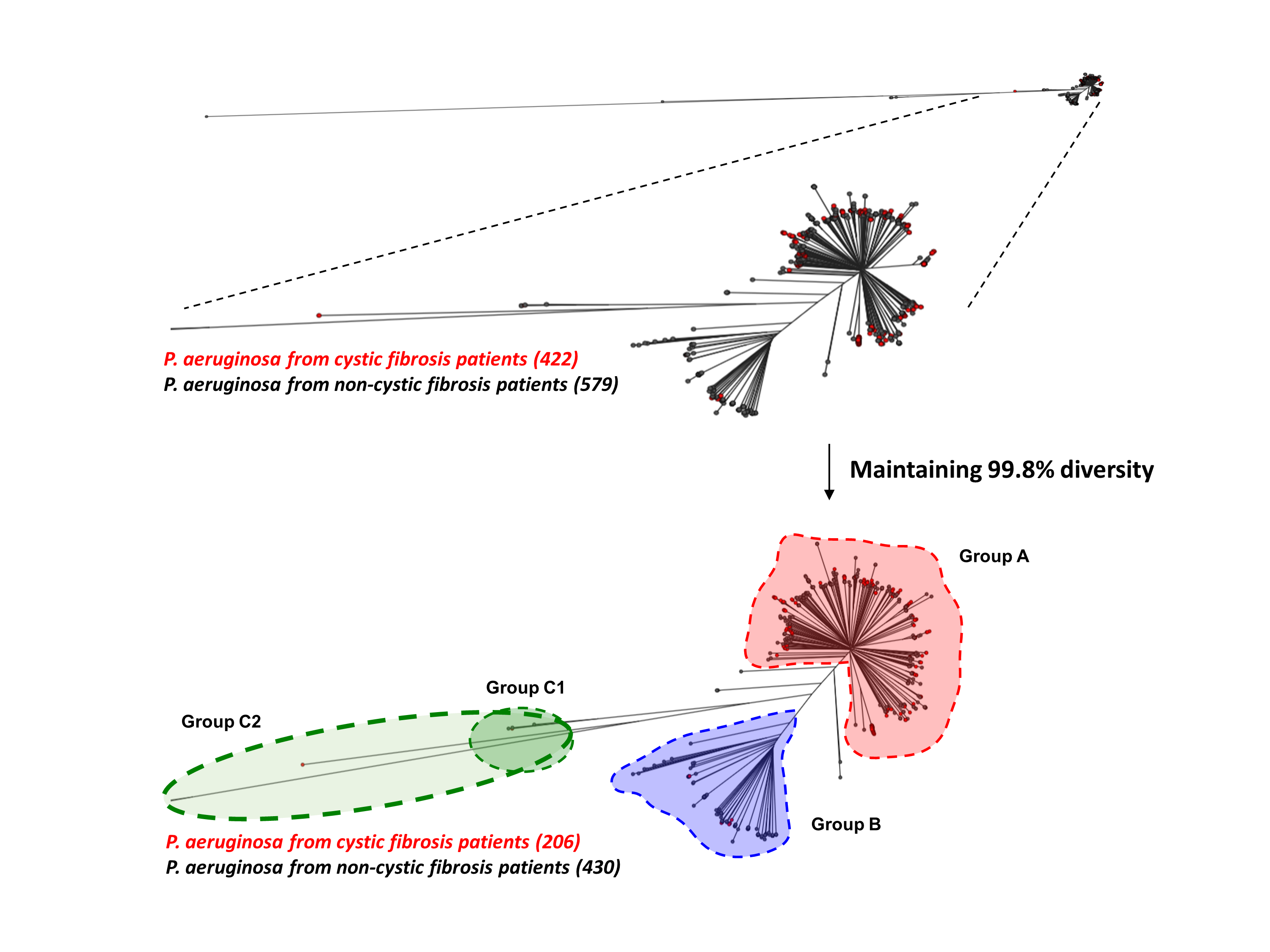

Supplement: S1 Fig — Upper phylogenetic tree was constructed with 1,001 genomes containing host disease information, and the tree below was drawn using 636 genomes and maintaining 99.8% diversity of the upper tree. Black and red leaves each indicate non-CF and CF isolates, and the numbers of CF and non-CF genomes for constructing each phylogenetic tree are placed inside brackets. (TIF) [file ppat.1009681.s001.TIF]

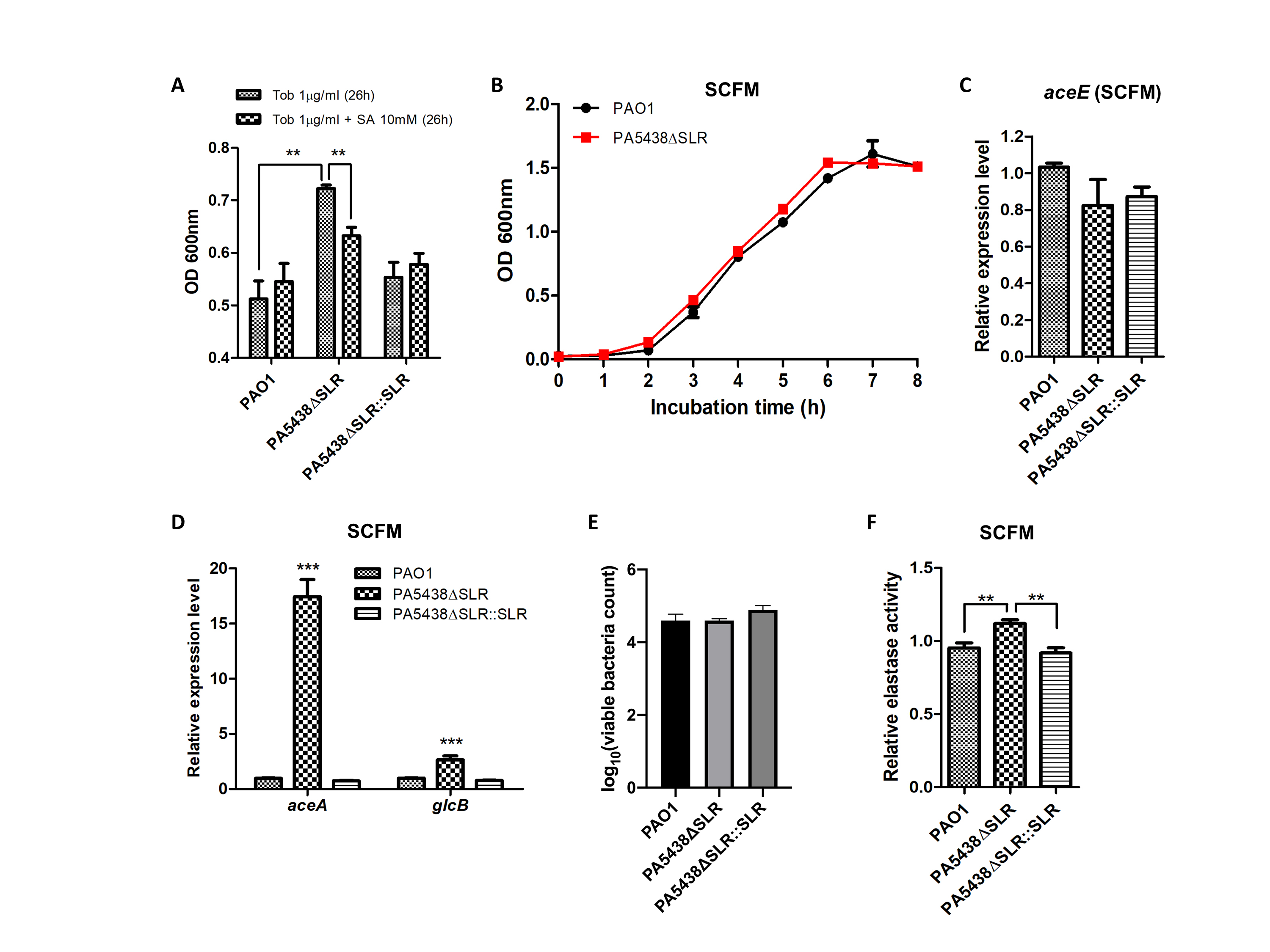

Supplement: S2 Fig — (A) Antibiotic susceptibility test with tobramycin (Tob) and Tob with 10 mM sodium acetate (SA) were performed. Initial OD600nm of PAO1, PA5438ΔSLR mutant, and the complementation strain were adjusted to 0.005, and OD600nm was measured after 26 hours of static incubation in LB supplemented with Tob. The concentration of antibiotic was 1 μg/ml. **p<0.01 (B) Growth curves of PAO1 and PA5438ΔSLR mutant in SCFM were observed over 8 hours. (C) RNAs of PAO1, the PA5438ΔSLR mutant, and the complementation strain were extracted at OD600nm ~0.9 in SCFM and relative expression levels of aceE were measured. (D) Relative expression levels of aceA and glcB of the same RNA used in (C) were measured. ***p<0.001 (E) The initial infection dosage was 107 CFU per 5 ✕ 105 neutrophils isolated from the bone-marrows of C57BL/6 mice. After 2 hours of co-culturing P. aeruginosa and neutrophils, we added gentamicin to the culture medium for 1 hour to remove any extracellular bacteria. We then harvested the intracellular bacteria by treating the neutrophils with 0.5% Triton-X, and measured the bacterial CFU. (F) After bacterial culture of PAO1, PA5438ΔSLR mutant, and the complementation strain in SCFM for 8 hours with shaking, an elastase assay was performed with culture supernatant. **p<0.01. (TIF) [file ppat.1009681.s002.TIF]

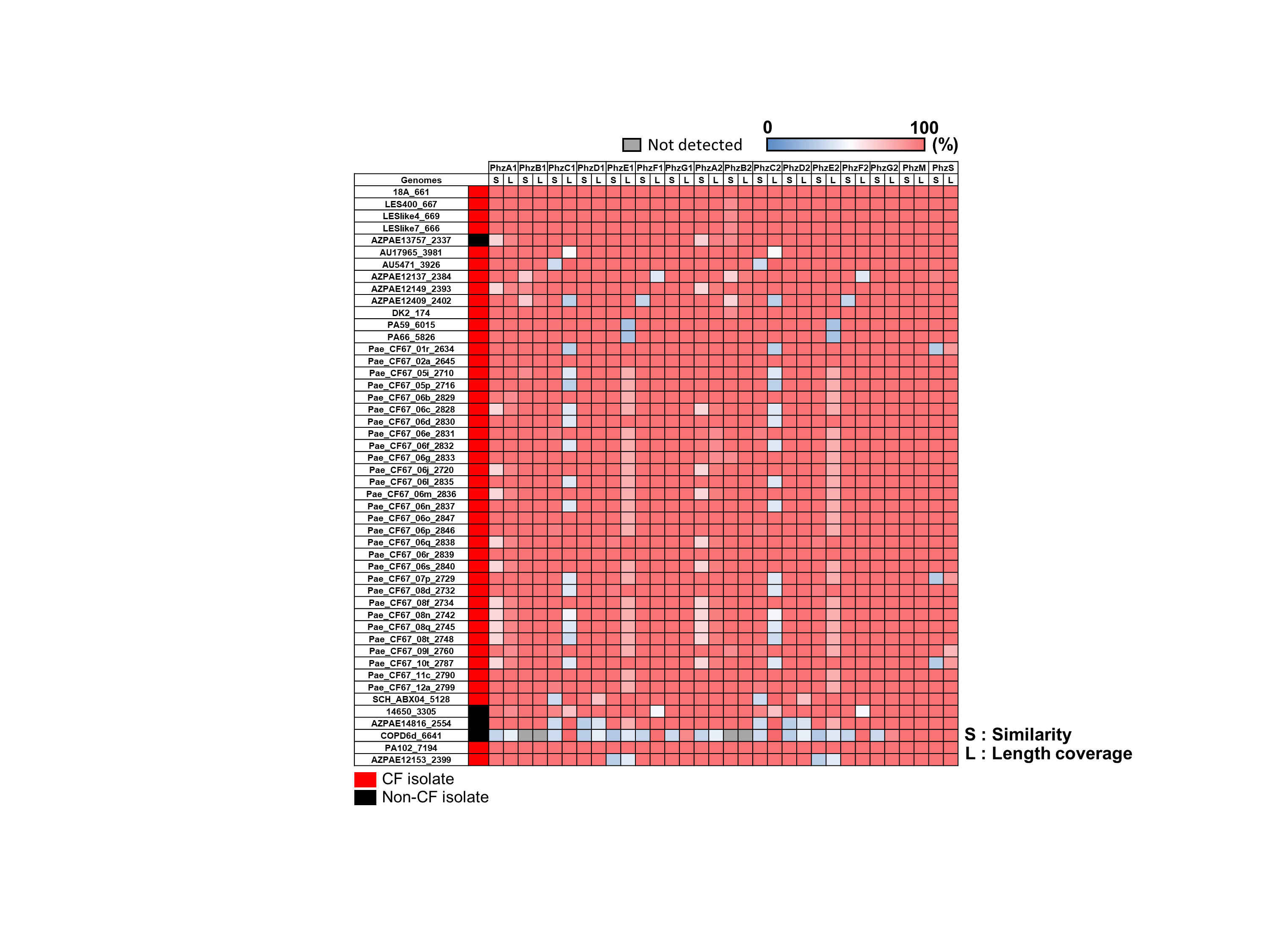

Supplement: S3 Fig — Each row represents a genome containing an SLR deletion in PA5438 homologs and color indicates whether it is included in CF or non-CF. Columns contain PAO1 proteins associated with pyocyanin biosynthesis and similarity (S), % of identical matches between reference PAO1 protein and its homolog, and length coverage (L), % of reference PAO1 protein sequence covered by its homolog. Gray box indicates there is no homolog under e-value 0.01 in the blastp search. (TIF) [file ppat.1009681.s003.TIF]

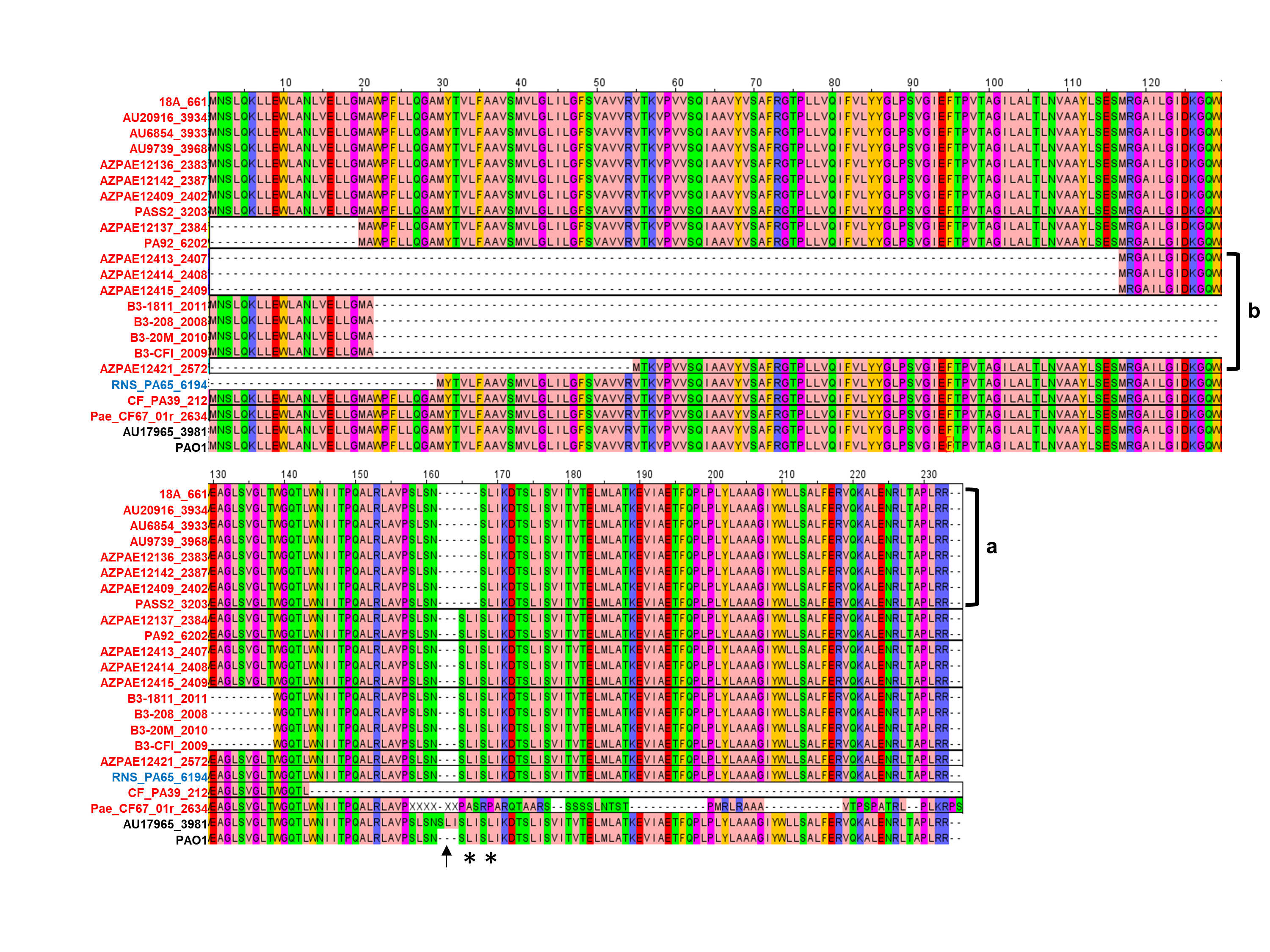

Supplement: S4 Fig — Multiple alignment of YecS and its homologs is visually represented. Names of the genomes are shown to the left of the multiple alignment. PAO1 and AU17965_3981 are representative genomes of non-CF and CF groups, and genomes labeled with red or blue belong to the CF or non-CF groups, respectively. Black arrow marks where the additional SLI insertion occurs (162nd to 164th residues) compared to the YecS protein. Regions marked by asterisks are regions of SLI amino acid repeat sequences in YecS. Genomes in a contain a deletion of SLI, resulting in a single copy of SLI, and genomes in b present large deletions in YecS. (TIF) [file ppat.1009681.s004.TIF]

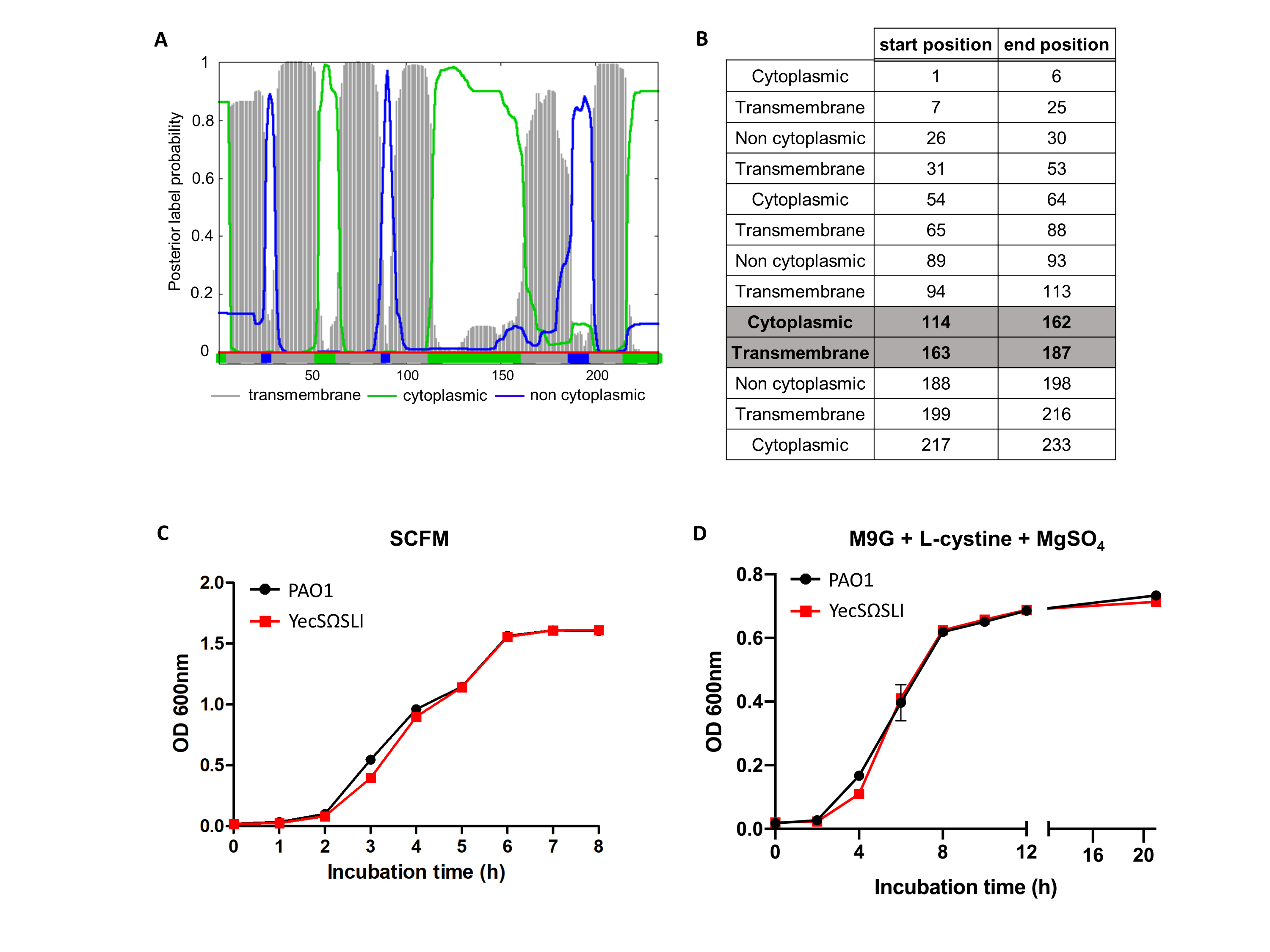

Supplement: S5 Fig — (A) Predicted transmembrane domains of the AU17965_3981_04951 protein are portrayed. Numbers below the figure indicate the amino acid loci. (B) Detailed amino acid ranges of the predicted transmembrane domains are listed. The SLI insertion in the AU17965_3981_4951 protein is present within the region highlighted in bold. (C) Growth curves of PAO1 and YecSΩSLI mutant in SCFM were measured over 8 hours. (D) Growth curves of PAO1 and YecSΩSLI in M9 minimal media supplemented with glucose, L-cystine, and MgSO4 were recorded over 21 hours. (TIF) [file ppat.1009681.s005.TIF]

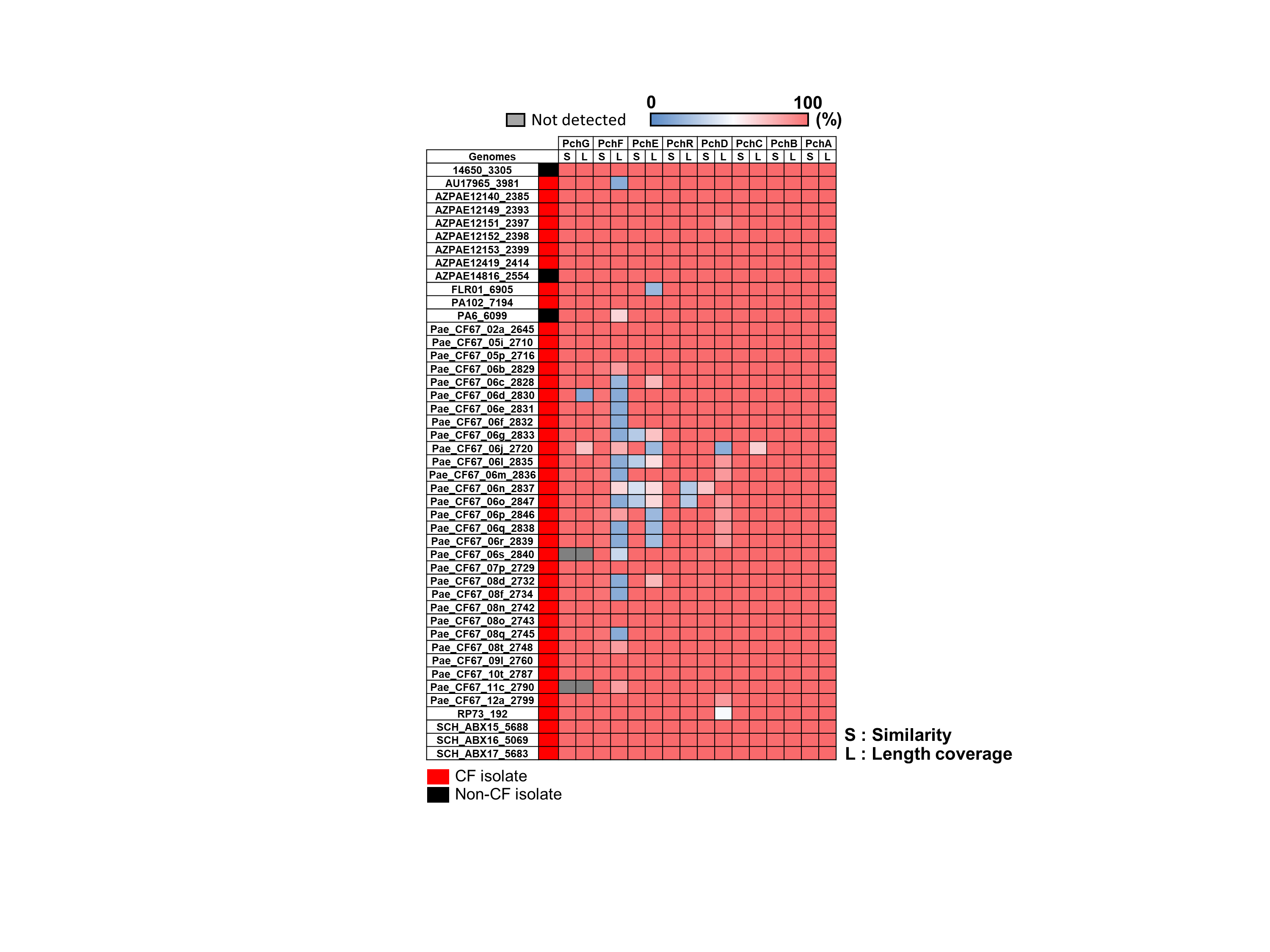

Supplement: S6 Fig — Each row represents a genome containing an SLI insertion in YecS homologs and color indicates whether it is included in CF or non-CF. Columns contain PAO1 proteins associated with pyochelin biosynthesis and similarity (S), % of identical matches between reference PAO1 protein and its homolog, and length coverage (L), % of reference PAO1 protein sequence covered by its homolog. Gray box indicates there is no homolog under e-value 0.01 in the blastp search. (TIF) [file ppat.1009681.s006.TIF]
