# Supplementary material for: Genome-wide association study of signature genetic alterations among pseudomonas aeruginosa cystic fibrosis isolates
Source: PLoS Pathog. 2021 Jun 23;17(6):e1009681. doi: 10.1371/journal.ppat.1009681 (PMC8274868; doi:10.1371/journal.ppat.1009681)
Supplement: S1 Table — (DOCX) [file ppat.1009681.s012.docx]

**Supplementary Materials for**

**Genome-Wide Association Study of Signature Genetic Alterations among *Pseudomonas aeruginosa* Cystic Fibrosis Isolates**

**This file includes:**

Table A to Table D

References

| **A** |  |  |  |  |  |  |  |
| --- | --- | --- | --- | --- | --- | --- | --- |
|  | **genomes** | **pident(%)** | **Length coverage(%)** |  | **genomes** | **pident(%)** | **Length coverage(%)** |
|  | 14650_3305 | 99.812 | 100 |  | Pae_CF67_06d_2830 | 99.812 | 100 |
|  | 18A_661 | 99.81 | 100 |  | Pae_CF67_06e_2831 | 99.812 | 100 |
|  | AU17965_3981 | 99.812 | 100 |  | Pae_CF67_06f_2832 | 99.812 | 100 |
|  | AU5471_3926 | 99.812 | 100 |  | Pae_CF67_06g_2833 | 99.812 | 100 |
|  | AZPAE12137_2384 | 99.812 | 100 |  | Pae_CF67_06j_2720 | 99.812 | 100 |
|  | AZPAE12149_2393 | 99.812 | 100 |  | Pae_CF67_06l_2835 | 99.812 | 100 |
|  | AZPAE12153_2399 | 99.812 | 100 |  | Pae_CF67_06m_2836 | 99.812 | 100 |
|  | AZPAE12409_2402 | 99.812 | 100 |  | Pae_CF67_06n_2837 | 99.812 | 100 |
|  | AZPAE13757_2337 | 99.812 | 100 |  | Pae_CF67_06o_2847 | 99.812 | 100 |
|  | AZPAE14816_2554 | 99.623 | 100 |  | Pae_CF67_06p_2846 | 99.812 | 100 |
|  | COPD6d_6641 | 99.812 | 100 |  | Pae_CF67_06q_2838 | 99.812 | 100 |
|  | DK2_174 | 99.812 | 100 |  | Pae_CF67_06r_2839 | 99.812 | 100 |
|  | LES400_667 | 99.812 | 100 |  | Pae_CF67_06s_2840 | 99.812 | 100 |
|  | LESlike4_669 | 99.812 | 100 |  | Pae_CF67_07p_2729 | 99.812 | 100 |
|  | LESlike7_666 | 99.812 | 100 |  | Pae_CF67_08d_2732 | 99.812 | 100 |
|  | PA102_7194 | 99.812 | 100 |  | Pae_CF67_08f_2734 | 99.812 | 100 |
|  | PA59_6015 | 99.812 | 100 |  | Pae_CF67_08n_2742 | 99.812 | 100 |
|  | PA66_5826 | 99.812 | 100 |  | Pae_CF67_08q_2745 | 99.812 | 100 |
|  | Pae_CF67_01r_2634 | 99.812 | 100 |  | Pae_CF67_08t_2748 | 99.812 | 100 |
|  | Pae_CF67_02a_2645 | 99.812 | 100 |  | Pae_CF67_09l_2760 | 99.812 | 100 |
|  | Pae_CF67_05i_2710 | 99.812 | 100 |  | Pae_CF67_10t_2787 | 99.812 | 100 |
|  | Pae_CF67_05p_2716 | 99.812 | 100 |  | Pae_CF67_11c_2790 | 99.812 | 100 |
|  | Pae_CF67_06b_2829 | 99.812 | 100 |  | Pae_CF67_12a_2799 | 99.812 | 100 |
|  | Pae_CF67_06c_2828 | 99.812 | 100 |  | SCH_ABX04_5128 | 99.812 | 100 |

| **B** |  |  |  |  |  |  |  |
| --- | --- | --- | --- | --- | --- | --- | --- |
|  | **genomes** | **pident(%)** | **Length**  **coverage(%)** |  | **genomes** | **pident(%)** | **Length**  **coverage(%)** |
|  | 14650_3305 | 99.862 | 100 |  | Pae_CF67_06d_2830 | 99.862 | 100 |
|  | 18A_661 | 99.72 | 100 |  | Pae_CF67_06e_2831 | 99.862 | 100 |
|  | AU17965_3981 | 99.862 | 100 |  | Pae_CF67_06f_2832 | 99.862 | 100 |
|  | AU5471_3926 | 99.862 | 100 |  | Pae_CF67_06g_2833 | 99.862 | 100 |
|  | AZPAE12137_2384 | 99.862 | 100 |  | Pae_CF67_06j_2720 | 99.862 | 100 |
|  | AZPAE12149_2393 | 99.862 | 100 |  | Pae_CF67_06l_2835 | 99.862 | 100 |
|  | AZPAE12153_2399 | 99.862 | 100 |  | Pae_CF67_06m_2836 | 99.862 | 100 |
|  | AZPAE12409_2402 | 99.862 | 100 |  | Pae_CF67_06n_2837 | 99.862 | 100 |
|  | AZPAE13757_2337 | 99.862 | 100 |  | Pae_CF67_06o_2847 | 99.862 | 100 |
|  | AZPAE14816_2554 | 99.724 | 100 |  | Pae_CF67_06p_2846 | 99.862 | 100 |
|  | COPD6d_6641 | 100 | 100 |  | Pae_CF67_06q_2838 | 99.862 | 100 |
|  | DK2_174 | 99.862 | 100 |  | Pae_CF67_06r_2839 | 99.862 | 100 |
|  | LES400_667 | 99.862 | 100 |  | Pae_CF67_06s_2840 | 99.862 | 100 |
|  | LESlike4_669 | 99.862 | 100 |  | Pae_CF67_07p_2729 | 99.862 | 100 |
|  | LESlike7_666 | 99.862 | 100 |  | Pae_CF67_08d_2732 | 99.862 | 100 |
|  | PA102_7194 | 99.862 | 100 |  | Pae_CF67_08f_2734 | 99.862 | 100 |
|  | PA59_6015 | 100 | 100 |  | Pae_CF67_08n_2742 | 99.862 | 100 |
|  | PA66_5826 | 100 | 100 |  | Pae_CF67_08q_2745 | 99.862 | 100 |
|  | Pae_CF67_01r_2634 | 99.862 | 100 |  | Pae_CF67_08t_2748 | 99.862 | 100 |
|  | Pae_CF67_02a_2645 | 99.862 | 100 |  | Pae_CF67_09l_2760 | 99.862 | 100 |
|  | Pae_CF67_05i_2710 | 99.862 | 100 |  | Pae_CF67_10t_2787 | 99.862 | 100 |
|  | Pae_CF67_05p_2716 | 99.862 | 100 |  | Pae_CF67_11c_2790 | 99.862 | 100 |
|  | Pae_CF67_06b_2829 | 99.862 | 100 |  | Pae_CF67_12a_2799 | 99.862 | 100 |
|  | Pae_CF67_06c_2828 | 99.862 | 100 |  | SCH_ABX04_5128 | 99.862 | 100 |

**Table A. Blastp results of AceA and GlcB.** Each column includes genomes (genomes containing SLR deletion in PA5438 homologs), pident (% of identical matches between reference PAO1 protein and its homolog), and length coverage (% of reference PAO1 protein sequence covered by its homolog). Black boxes indicate non-CF genomes and red boxes indicate CF genomes. **(A)** Blastp results were conducted with the PAO1 AceA and its homologs from genomes containing SLR deletion in the PA5438 homologs. **(B)** Blastp results were conducted with the PAO1 GlcB and its homologs from genomes containing SLR deletion in the PA5438 homologs.

| **A** |  |  |  |  |  |
| --- | --- | --- | --- | --- | --- |
|  | **genomes** | **pident(%)** |  | **genomes** | **pident(%)** |
|  | 14650_3305 | 100 |  | Pae_CF67_06d_2830 | 100 |
|  | 18A_661 | 99.5 |  | Pae_CF67_06e_2831 | 100 |
|  | AU17965_3981 | 100 |  | Pae_CF67_06f_2832 | 100 |
|  | AU5471_3926 | 100 |  | Pae_CF67_06g_2833 | 100 |
|  | AZPAE12137_2384 | 100 |  | Pae_CF67_06j_2720 | 100 |
|  | AZPAE12149_2393 | 100 |  | Pae_CF67_06l_2835 | 100 |
|  | AZPAE12153_2399 | 100 |  | Pae_CF67_06m_2836 | 100 |
|  | AZPAE12409_2402 | 99.5 |  | Pae_CF67_06n_2837 | 100 |
|  | AZPAE13757_2337 | 100 |  | Pae_CF67_06o_2847 | 100 |
|  | AZPAE14816_2554 | 99.5 |  | Pae_CF67_06p_2846 | 100 |
|  | COPD6d_6641 | 100 |  | Pae_CF67_06q_2838 | 100 |
|  | DK2_174 | 100 |  | Pae_CF67_06r_2839 | 100 |
|  | LES400_667 | 100 |  | Pae_CF67_06s_2840 | 100 |
|  | LESlike4_669 | 100 |  | Pae_CF67_07p_2729 | 100 |
|  | LESlike7_666 | 100 |  | Pae_CF67_08d_2732 | 100 |
|  | PA102_7194 | 100 |  | Pae_CF67_08f_2734 | 100 |
|  | PA59_6015 | 100 |  | Pae_CF67_08n_2742 | 100 |
|  | PA66_5826 | 100 |  | Pae_CF67_08q_2745 | 100 |
|  | Pae_CF67_01r_2634 | 100 |  | Pae_CF67_08t_2748 | 100 |
|  | Pae_CF67_02a_2645 | 100 |  | Pae_CF67_09l_2760 | 100 |
|  | Pae_CF67_05i_2710 | 100 |  | Pae_CF67_10t_2787 | 100 |
|  | Pae_CF67_05p_2716 | 100 |  | Pae_CF67_11c_2790 | 100 |
|  | Pae_CF67_06b_2829 | 100 |  | Pae_CF67_12a_2799 | 100 |
|  | Pae_CF67_06c_2828 | 100 |  | SCH_ABX04_5128 | 100 |

| **B** |  |  |  |  |  |
| --- | --- | --- | --- | --- | --- |
|  | **genomes** | **pident(%)** |  | **genomes** | **pident(%)** |
|  | 14650_3305 | 100 |  | Pae_CF67_06d_2830 | 100 |
|  | 18A_661 | 100 |  | Pae_CF67_06e_2831 | 100 |
|  | AU17965_3981 | 99.5 |  | Pae_CF67_06f_2832 | 100 |
|  | AU5471_3926 | 100 |  | Pae_CF67_06g_2833 | 100 |
|  | AZPAE12137_2384 | 100 |  | Pae_CF67_06j_2720 | 100 |
|  | AZPAE12149_2393 | 100 |  | Pae_CF67_06l_2835 | 100 |
|  | AZPAE12153_2399 | 100 |  | Pae_CF67_06m_2836 | 100 |
|  | AZPAE12409_2402 | 100 |  | Pae_CF67_06n_2837 | 100 |
|  | AZPAE13757_2337 | 100 |  | Pae_CF67_06o_2847 | 100 |
|  | AZPAE14816_2554 | 100 |  | Pae_CF67_06p_2846 | 100 |
|  | COPD6d_6641 | 100 |  | Pae_CF67_06q_2838 | 100 |
|  | DK2_174 | 100 |  | Pae_CF67_06r_2839 | 100 |
|  | LES400_667 | 100 |  | Pae_CF67_06s_2840 | 100 |
|  | LESlike4_669 | 100 |  | Pae_CF67_07p_2729 | 100 |
|  | LESlike7_666 | 100 |  | Pae_CF67_08d_2732 | 100 |
|  | PA102_7194 | 100 |  | Pae_CF67_08f_2734 | 100 |
|  | PA59_6015 | 100 |  | Pae_CF67_08n_2742 | 100 |
|  | PA66_5826 | 100 |  | Pae_CF67_08q_2745 | 100 |
|  | Pae_CF67_01r_2634 | 100 |  | Pae_CF67_08t_2748 | 100 |
|  | Pae_CF67_02a_2645 | 100 |  | Pae_CF67_09l_2760 | 100 |
|  | Pae_CF67_05i_2710 | 100 |  | Pae_CF67_10t_2787 | 100 |
|  | Pae_CF67_05p_2716 | 100 |  | Pae_CF67_11c_2790 | 100 |
|  | Pae_CF67_06b_2829 | 100 |  | Pae_CF67_12a_2799 | 100 |
|  | Pae_CF67_06c_2828 | 100 |  | SCH_ABX04_5128 | 99.5 |

**Table B. Blastn results of promoter regions (upstream 200 bp) of *aceA* and *glcB*.** Columns parallel to those in S1 Table. **(A)** Blastn results were conducted with the *aceA* promoter region in the PAO1 genome and its homologous promoters from genomes containing SLR deletion in the PA5438 homologs. **(B)** Blastn results were conducted with the *glcB* promoter region in the PAO1 genome and its homologous promoters from genomes containing SLR deletion in the PA5438 homologs.

| **Bacterial strains** **and plasmids** | **Genotype or description** | **Reference** **/source** |
| --- | --- | --- |
| ***E. coli* strains** |  |  |
| DH5αλ*pir* | *fhuA2 lac(del)U169 phoA glnV44* Φ*80*′ *lacZ(del)M15 gyrA96 recA1 relA1 endA1 thi-1 hsdR17* λ*pir* |  |
| SM10λpir | thi thr leu tonA lacY supE recA::RP4-2-Tc::Mu Kmr λpir |  |
| ***P. aeruginosa* strains** |  |  |
| PAO1 | WT, laboratory strain of *P. aeruginosa* | This study |
| △*pch*△*pvd* | Pyochelin and pyoverdine synthesis-defective PAO1 mutant | Ref. 63 |
| YecSΩSLI | WT with SLI amino acids insertion in 162nd residue of YecS | This study |
| YecSΩSLI::△SLI | YecSΩSLI with SLI amino acids deletion from 162nd to 164th residues of YecS | This study |
| PA5438△SLR | WT with SLR amino acids deletion from 272nd to 274th residues of PA5438 | This study |
| PA5438△SLR::SLR | PA5438△SLR with SLR amino acids insertion in 272nd residue of PA5438 | This study |
| con::*lacZ* | WT with puc18-mini-Tn7t-Gm-lacZ | This study |
| *phuR*::*lacZ* | WT with puc18-mini-Tn7t-Gm-lacZ containing the intergenic region between phuR and phuS in front of lacZ | This study |
| *phuR*C117T::*lacZ* | WT with puc18-mini-Tn7t-Gm-lacZ containing the intergenic region between phuR and phuS with snp from cytosine to thymine at 117th residue in front of lacZ | This study |
| **Plasmids** |  |  |
| pCVD442 | Amp^r,^ Gm^r^ suicide vector containing *sacB* for screening recombinant | This study |
| puc18-mini-Tn7t-Gm-*lacZ* | Amp^r^, Gm^r^, site-specific chromosomal insertion plasmid harboring promoterless *lacZ* | Ref. 79 |
| pTNS2 | Helper plasmid containing site-specific recombinase for chromosomal insertion | Ref. 79 |

**Table C. Strains and plasmids used in this study.**

| **Primers** | **Sequence (5′–3′)** | **Description** |
| --- | --- | --- |
| yecS#1 | TATTCCCGGGAAGTGGTTCGGCCTGGACGT | Left-flanking forward primer of *yecS* containing XmaI restriction enzyme site |
| yecS#2 | ATCAGGCTGTTCGACAGGCTCGGCACCG | Left-flanking reverse primer of *yecS* containing reverse complementary sequence of CAGCCTGAT (SLI) |
| yecS#3 | GATCAGGCTGATCAGGCTGTTCGACAGGC | Left-flanking reverse primer of *yecS* containing overlapping site to yecS#4 |
| yecS#4 | CAGCCTGATCAGCCTGATCAGCCTGATCA | Right-flanking forward primer of *yecS* containing CAGCCTGAT (SLI) sequence |
| yecS#5 | GCCTGTCGAACAGCCTGATCAGCCTGATC | Right-flanking forward primer of *yecS* containing overlapping site to yecS#2 |
| yecS#6 | TATTGAGCTCTTCGACCTGCTCTCGCGCT | Right-flanking reverse primer of *yecS* containing SacI  restriction enzyme site |
| PA5438#1 | TATTCCCGGGAAGCTGAAGCTGGCGCAGAG | Left-flanking forward primer of SLR of PA5438 at 272^nd^ containing XmaI restriction enzyme site |
| PA5438#2 | GGCGCAGGCTGCGCTTGACGCTCTTGAGGT | Left-flanking reverse primer of SLR of PA5438 at 272^nd^ containing overlapping site to PA5438#3 |
| PA5438#3 | CGTCAAGCGCAGCCTGCGCCTGTCGCC | Right-flanking forward primer of SLR of PA5438 at 272^nd^ containing overlapping site to PA5438#2 |
| PA5438#4 | TATTGAGCTCGAGATCGCCTCGCTGGAGT | Right-flanking reverse primer of SLR of PA5438 at 272^nd^ containing SacI restriction enzyme site |
| phuR_phuS#1 | TATTCTCGAGCGACGGTTCCTCGGGCATAT | Forward primer of intergenic region between *phuR* and *phuS* containing XhoI restriction enzyme site |
| phuR_phuS#2 | TATTAAGCTTGTGGGACTCCTTGGGTCGG | Reverse primer of intergenic region between *phuR* and *phuS* containing HindIII restriction enzyme site |
| aceA_RT#1 | GACCTGCTCTGGATCGAAAC | *aceA* forward primer for qPCR |
| aceA_RT#2 | TCAGGGTCCAGTTGAAGGAC | *aceA* reverse primer for qPCR |
| glcB_RT#1 | ACATCCTGACCATTCCGCTG | *glcB* forward primer for qPCR |
| glcB_RT#2 | GGACGACATAGCCGAGGATG | *glcB* reverse primer for qPCR |
| aceE_RT#1 | CCAAGACCATCAAGGGCTAC | *aceE* forward primer for qPCR |
| aceE_RT#2 | ACCGGGATGTCGAACTTGTC | *aceE* reverse primer for qPCR |
| 16srDNA#1 | CTTACGGCCAGGGCTACACA | 16S rRNA forward primer for qPCR |
| 16srDNA#2 | GTACAAGGCCCGGGAACGTA | 16S rRNA reverse primer for qPCR |

**Table D. Primers used in this study.**

**Supporting Information**

**Table A. Blastp results of AceA and GlcB.** Each column includes genomes (genomes containing SLR deletion in PA5438 homologs), pident (% of identical matches between reference PAO1 protein and its homolog), and length coverage (% of reference PAO1 protein sequence covered by its homolog). Black boxes indicate non-CF genomes and red boxes indicate CF genomes. **(A)** Blastp results were conducted with the PAO1 AceA and its homologs from genomes containing SLR deletion in the PA5438 homologs. **(B)** Blastp results were conducted with the PAO1 GlcB and its homologs from genomes containing SLR deletion in the PA5438 homologs.

**Table B. Blastn results of promoter regions (upstream 200 bp) of *aceA* and *glcB*.** Columns parallel to those in S1 Table. **(A)** Blastn results were conducted with the *aceA* promoter region in the PAO1 genome and its homologous promoters from genomes containing SLR deletion in the PA5438 homologs. **(B)** Blastn results were conducted with the *glcB* promoter region in the PAO1 genome and its homologous promoters from genomes containing SLR deletion in the PA5438 homologs.

**Table C. Strains and plasmids used in this study.**

**Table D. Primers used in this study.**
